# Nonlinear backbone torsional pair correlations in proteins

**DOI:** 10.1038/srep34481

**Published:** 2016-10-06

**Authors:** Shiyang Long, Pu Tian

**Affiliations:** 1 School of Life Sciences, Jilin University, Changchun, 130012 China; 2 MOE Key Laboratory of Molecular Enzymology and Engineering, Jilin University, Changchun, 130012 China

## Abstract

Protein allostery requires dynamical structural correlations. Physical origin of which, however, remain elusive despite intensive studies during last two and half decades. Based on analysis of molecular dynamics (MD) simulation trajectories for ten proteins with different sizes and folds, we found that nonlinear backbone torsional pair (BTP) correlations, which are mainly spatially long-ranged and are dominantly executed by loop residues, exist extensively in most analyzed proteins. Examination of torsional motion for correlated BTPs suggested that such nonlinear correlations are mainly associated aharmonic torsional state transitions and in some cases strongly anisotropic local torsional motion of participating torsions, and occur on widely different and relatively longer time scales. In contrast, correlations between backbone torsions in stable *α* helices and *β* strands are mainly linear and spatially short-ranged, and are more likely to associate with harmonic local torsional motion. Further analysis revealed that the direct cause of nonlinear contributions are heterogeneous linear correlations. These findings implicate a general search strategy for novel allosteric modulation sites of protein activities.

Allostery in protein molecules is defined by their response to external stimuli on distal site(s). Most biologically relevant allostery are spatially long-ranged (SLR)^1^,^2^,^3^,^4^,^5^. Therefore, understanding of structural correlations, especially SLR ones, are essential for elucidation and manipulation of protein allostery. Earlier computational characterization of dynamical correlations^6^,^7^,^8^ provided insightful information on the inherent correlated motion of proteins on nanoseconds and shorter time scales. Li *et al*.^9^ analyzed a 700-*ns* molecular dynamics (MD) simulation trajectory of ubiquitin and concluded that SLR pair correlations are rather rare and network of short-ranged coherent motions likely contribute to transmission of information in allostery. By combining NMR and computational ensemble, Fenwick *et al*.^10^ concluded that the observed SLR correlations in ubiquitin are likely to be transmitted by network of hydrogen bonds. Along the same line, Fenwick *et al*.^11^ provided evidence that hydrogen bonds across *β*-sheets mediate concerted motions, which are candidates for transfer of structural information over relatively long distances. Papaleo *et al*.^12^ combined dynamical cross-correlation and a description of the protein as a network of interacting residues to detect communication pathways from MD simulation trajectories of the E2 enzyme. In these studies, analyses were limited to linear correlations^6^,^7^,^8^,^9^,^10^,^11^,^12^. It was well-recognized that nonlinear correlations exist in protein dynamics and a generalized correlation measure was developed to be within the range of [0, 1] based on nonlinear transformation of mutual information (MI)^13^. A procedure of mutual information based correlation analysis was developed and utilized to identify SLR correlations in human interleukin-2^14^. However, despite important insight revealed in these studies, the physical origin and underlying molecular motions of observed correlations remain elusive. In this study, we focus on molecular motions that underly backbone torsional pair (BTP) correlations. After calculating both mutual information and linear correlations for BTPs in extensive MD simulation trajectories of ten proteins with different sizes and folds (Fig. 1), we analyzed variation of correlations as a function of sequential and spatial distances, of belonging secondary structures, and of torsional motions and time scales. It was found that linear correlations of BTPs are predominantly spatially short-ranged, mainly associate with harmonic/isotropic local torsional motions and occur on relatively short time scales. On the other hand, nonlinear correlations occur for both spatially short and long-ranged BTPs, they mainly associate with aharmonic torsional state transitions on widely different and relatively longer time scales, and are dominantly executed by loop residues. The direct cause of nonlinear BTP correlations are found to be heterogeneous linear correlations associated with different torsional states or strongly anisotropic local motion of participating torsions.

## Results

### Mutual information and linear correlations of BTPs

Based on the full correlation expansion as derived by Matsuda^15^ (Equation (2.10) in the reference and shown below),


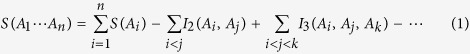


(*S* represent informational entropy of a joint ensemble *A*_1_ through *A*_*n*_, with *I*_2_ being the second order mutual information and *I*_*j*_ being the general *j*th order mutual information), it is evident, and has been well recognized^14^,^16^,^17^, that mutual information is an inherent component of entropy, thus is intimately related to free energy at a given temperature. Therefore, utilization of mutual information to characterize dynamical correlations makes more energetic sense than both linear correlations or the generalized correlation. However, it remains unclear how linear correlations relate to mutual information, and consequently entropy and free energy in proteins. To elucidate this issue, we calculated second order mutual information (*MI*) and linear correlation coefficient *r* (see *Methods* in supporting info for details) for all pairs of backbone dihedrals *ϕ* and *ψ* for ten protein molecules. *MI* vs. *r* plots of four proteins were presented in Fig. 2 (and Fig. S10 for similar plots of the remaining 6 proteins). Contour lines of these scatter plots approximately reflect relationship (see *Methods* for details) between *r* and maximum possible *MI* (denoted *MPMI*_*r*_ here after) engendered by corresponding linear correlations (as there is always possibility of nonlinear correlations for any given BTP). It is found that contour lines are essentially the same regardless of the identity of proteins, and may be reasonably well-fit (Figs 2 and S10) with the following function. This equation is an empirical fitting that applies well to all data. The major considerations are i) symmetry for the two sides of the minimum and ii) the asymptotic value of mutual information goes from 0 to ∞, which is consistent with a logarithm function defined on the domain [1, ∞ ]:


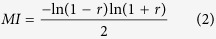


Meanwhile, data points locate above and far from the contour line (as specified by Equation 2) indicate that significant nonlinear correlations exist for corresponding BTPs. Again, note that *MI* is linearly related to entropy by the Boltzmann constant. Therefore, for points fall on Equation 2, entropic cost for initial increase of *r* from 0.1 to 0.4 is around 0.08*k*_*B*_, while a further increase of *r* from 0.4 to 0.7 corresponds to approximately 0.23*k*_*B*_. In extreme cases, one may have large variation of linear correlations with no thermodynamic impact at all, this can be easily imagined if one draw a horizontal line in one of *MI* vs. *r* plots as shown in Fig. 2a. Therefore, utilization of linear correlations to characterize protein torsional correlations does not provide thermodynamic support (see supporting text *Complex mapping between mutual information and linear correlations* and relevant figures (Figs S2–S9) for more discussions). Additionally, while the theoretical range of *MI* goes from 0 to ∞, thermodynamics dictates that we will not observe huge values in practical biomolecular systems, which operate under ambient conditions. Indeed, as shown in Fig. 2 (and Fig. S10), the maximum *MI* we observed is less than 1.3 and *MI* value beyond 1.0 is extremely rare. Based on these analysis and observations, we concluded that utilization of *MI* to characterize dynamical correlations provides both practical convenience and physical intuition. Nonetheless, we analyzed linear correlations extensively for comparison with *MI* on the one hand, and to identify nonlinear contributions to BTP correlations on the other hand.

### Sequential distribution of BTP correlations

To analyze the distribution of both linear correlations and *MI* of BTPs in primary sequence space, a correlation matrix was created for each analyzed protein and presented in Fig. 3 (and Fig. S11). For convenience of presentation on the same matrix, *r* was first transformed into *MPMI*_*r*_ by utilizing Equation (2). For sequentially long-ranged pairs (off diagonal points in correlation matrices), the full *MI* (presented in left-upper half matrices), which includes both linear and nonlinear contributions, is significantly larger than *MPMI*_*r*_ (presented in right-lower half matrices) in most of proteins analyzed. The observation suggests that nonlinear contributions are increasingly more important over longer distances in primary sequences. However, the extent of difference between full *MI* and *MPMI*_*r*_ varies significantly for different proteins, and range from non-significant for the two smallest proteins (1*bta*, 5*pti*) to dramatic for larger proteins (see Fig. 3d, Figs S11d and S11ae). Additionally, a common feature shared by all proteins is that significant *MI* in off-diagonal region is primarily associated with loop residues (all residues that are in neither an *α* helix nor a *β* strand were defined as loop residues in this study).

### Relevance of spatial distances and secondary structures for nonlinear BTP correlations

In three dimensional protein structures, large distances in primary sequence may correspond to either long or short distances in space. Correlations caused by physical adjacency are trivially expected in condensed phases. In practice, what we care most are SLR correlations due to their potential participation in functionally important allosteric interactions. To analyze spatial variance of BTP correlations, the calculated *MI* and *MPMI*_*r*_ were plotted with respect to spatial distances as shown in Fig. 4 (and in Fig. S12). Two major consistent features were observed in all of studied proteins. Firstly, SLR correlations have significant nonlinear contributions since *MI* are generally larger than corresponding *MPMI*_*r*_. Secondly, loop-loop (*L*-*L*) BTPs exhibit the most and the strongest, *α* helix and *β* strand (*α*/*β*-*α*/*β*) BTPs have the least and the weakest, and *α*/*β*-loop (*α*/*β*-L) BTPs manifest intermediate SLR correlations. Regarding the second feature, significant variation was observed among different proteins (e.g. 3*f*3*y* exhibits significantly more extensive SLR *L*-*L* BTP correlations than 1*bta*, Fig. S12ad).

Qualitatively, correlation matrices for studied proteins (see Figs 3 and S11) suggest that for significant sequentially non-local correlations (off-diagonal region), nonlinear contributions are significant. Similarly, distance vs. correlation plots in Fig. 4 (and Fig. S12) indicate that SLR correlations have significant nonlinear contributions and this is especially true for some *L*-*L* BTPs. To further clarify relative importance of nonlinear correlations for different types of BTPs (i.e. *L*-*L*, *α*/*β*-*L* and *α*/*β*-*α*/*β*) at different spatial distances, we constructed *MI* vs. *r* plots for spatially local (with inter-torsion distances equal to or smaller than 8 Å) and non-local (otherwise) BTPs of each type and presented the results in Fig. 5 (and Fig. S13). For most proteins, *α*/*β*-*α*/*β* BTPs exhibit extremely rare (except for 3*f*3*y*) nonlinear correlations that locate above the indicated contour line specified by Equation 2, *L*-*L* BTPs have the most number of data points exhibit significant nonlinear correlations, and *α*/*β*-*L* BTPs stays in between. The relative ratio of BTPs with significant long-range correlations were listed for *L*-*L*, *L*-*α*/*β*, and *α*/*β*-*α*/*β* types for each protein in Table S3. Spatial locality, while makes decisive difference in correlation strength, plays a unimportant role in relative significance of linear and nonlinear contributions among different types of BTPs. Human sulfertransferase seems to be an outlier with significant proportion of *α*/*β*-*α*/*β* BTPs locate above the contour line (Fig. S13d). Nonetheless, even for this seemingly special protein, spatial locality remains to be an non-decisive factor in determining relative importance of nonlinear contributions in correlations of different types of BTPs (*α*/*β*-*α*/*β*, *α*/*β*-*L* and *L*-*L*). It is important to note that, in all studied proteins, the majority BTPs fall on or locate closely to the contour line in *MI* vs. *r* plots (Fig. 5) regardless of specific BTP types (Table 1). Therefore, linear correlation contributes dominantly for most of BTPs irrespective of the specific secondary structures in which the participating torsion locate. It is only that *L*-*L* BTPs are the most likely, and *α*/*β*-*α*/*β* BTPs are the least likely to have significant nonlinear contributions to their correlations, with *α*/*β*-*L* BTPs being the intermediate scenario in this regard.

### Torsional state transitions and nonlinear BTP correlations

Based on observations mentioned above, we were quite confident that neither spatial distance nor the specific identity of belonging secondary structure is a necessary factor for significant nonlinear contributions in BTP correlations. Instead, it should be some other property that is the most likely to associate with loop residues and is the least likely to associate with residues in stable secondary structures. For backbone torsions of loop residues, one outstanding feature is significantly higher probability of having torsional state transitions on various time scales. In contrast, most backbone torsions in stable secondary structures stay in one specific torsional state for native proteins. To test for necessity of torsional state transitions in nonlinear contributions to BTP correlations, we calculated distributions of all *ϕ*s and *ψ*s for each of studied proteins and searched for torsional state transitions according to the specified rule (see *Methods*-*Distributions of backbone torsions and joint distributions for BTPs* in supporting info for details), and the results were shown in Table 1. Indeed, a nonlinearly correlated BTP (see “Nonlinear BTPs” column in Table 1) belongs the most likely to the “DMP (double multiple peak)” scenario, the least likely to the “DSP (double single peak)” scenario, and with the intermediate probability belonging to the “SMP (single multiple peak)” case. Again, it is important to note that regardless of the torsional state transition status, most BTPs are not significantly correlated and such BTPs were classified as “Linear BTPs” in Table 1. Since joint distributions of a given BTP is not directly deducible from distributions of its participating torsions, we proceeded to search for peaks (local maxima) of the joint distribution of each BTP (see *Methods*-*Distributions of backbone torsions and joint distributions for BTPs* in supporting info for details). The results were shown in Fig. 6 (and Fig. S14). It is observed that the contour line is mainly covered by BTPs with single-peak joint distributions. The majority of BTPs locate far above the contour line have three or more peaks that are associated with torsional state transitions in participating torsions. However, there are small but significant number of BTPs with single joint peak distributions locate above the contour line, and there are rare BTPs with double or triple-peak joint distributions fall onto or locate slightly below the contour line. Therefore, while single-peak distributions of participating torsions (or BTP joint distributions) associate predominantly with linear correlations and multiple-peak distributions associate predominantly with nonlinear contributions, the small number of exceptions suggested that torsional state transitions are neither necessary nor sufficient conditions for, but associate significantly more tightly with nonlinear BTP correlations than sequential distances, spatial distances and identity of belonging secondary structures. Additionally, for spatially local BTPs (Figs 6a–d and S14a–f), a large fraction of points fall onto or locate close to the contour line exhibit significant linear correlations, while most of data points fall onto or near the contour line have weak linear correlations for SLR BTPs (Figs 6e–h and S14g–l). These observations further suggest that it is quite difficult for linear correlations to propagate over long distances spatially in proteins.

### Nonlinear contributions and heterogeneous linear correlations

BTPs that locate above the contour line but with both participating torsions exhibiting single peak distributions (varies from 0.4% for 3*f*3*y* to 15% for 5*pti*, Table 1) suggested that nonlinear contribution to BTP correlation may exist independent of torsional state transitions. These BTPs also exhibit single peak joint distributions (Fig. 6). Conversely, there are also rare BTPs that both fall on the contour line and have double- or triple-peak joint distributions and corresponding multiple-peak distributions for one participating torsions corresponding to torsional state transitions. Such observations indicate that predominantly linear correlations may exist for BTPs with participating torsions having torsional state transitions. We are interested in pursuing the origin of nonlinear contributions that are independent of torsional state transitions in the former case (termed *single-peak nonlinear case* below), and the reason why torsional state transitions in later cases resulted in negligible nonlinear contributions (termed *multiple-peak linear case* below).

For two independent DOFs *x* and *y*, their joint distribution is given as:


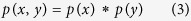


the effective correlation between two correlated variables *x* and *y* should be reflected by the following distribution difference:


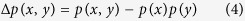


Therefore, analysis of these two distributions for corresponding BTPs may help us reveal physical mechanisms behind both the *single-peak nonlinear case* and *multiple-peak linear case*.

We first examined BTPs belong to the *single-peak nonlinear case*, and found that their joint distributions are continuous with various highly anisotropic shape, the overwhelming majority of which have either two elliptical joint distribution peaks in immediate contact or a “L” shape with various extent of splay (see Fig. 7c1), and Fig. 7b1)), and both scenarios correspond to strongly anisotropic motion of participating torsions within a single torsional state. In contrast, for typical BTPs with single-peak joint distributions and fall on the contour line, the joint distribution peak is approximately ellipse with various eccentricity (Fig. 7a1)). More importantly, in the distribution difference plots, at least one set of same-signed peaks (but lines were drawn for positvely-signed peaks only) fall approximately on the same straight line for linearly correlated BTPs (Fig. 7a2a3)), which suggested that linear correlations for all observed data are approximately homogeneous. In contrast, two or more line segments with different slopes are necessary to connect same-signed peaks for *single-peak nonlinear case* (Fig. 7b2,b3), Fig. 7c2,c3)), which suggested that heterogeneous linear correlations exist for different subpart of the observed data. Additionally, for BTPs fall on the bottom part of the contour line, the participating torsions are essentially independent and no peak exist for the distribution difference (Fig. 7d2)), and consequently no lines may be drawn (Fig. 7d3)).

We further selected BTPs belong to the *multiple-peak linear case* (Fig. 7e1)) and a few other representative BTPs with multiple-peak joint distributions (that correspond to torsional state transitions of participating torsions) and significant nonlinear contributions in their pair correlations (Fig. 7f1,g1,h1)), and examined corresponding joint distributions and distribution differences. It was found that for BTPs locate far above the contour line, multiple line segments with different slopes are necessary to connect same-signed peaks in distribution difference(Fig. 7f2,f3,g2,g3,h2,h3)), which suggested that linear correlations between the two participating torsions are highly heterogeneous. Consequently, correlations for such BTPs may not be properly represented by a single linear correlation coefficient and mutual information is a better choice. While for BTPs fall onto or locate in the vicinity of the contour line, same-signed peaks falls approximately on a single straight line, which suggested that linear correlations for such BTP is essentially homogeneous (see Fig. 7e2,e3)).

These observations demonstrate that regardless of torsional state transitions, as long as a pair of DOFs have heterogeneous linear correlations, it is likely that there are significant nonlinear contributions to their pair correlation. The reason is that it is not possible to effectively represent such heterogeneous linear correlations with a single linear correlation coefficient. It is noted that heterogeneous linear correlations do not guarantee significant nonlinear correlation. Combinations of heterogeneous linear correlations may result in effectively negligible global correlations. Therefore, a safe description is that for a pair of significantly correlated DOFs, heterogeneous linear correlations are both necessary and sufficient for nonlinear contributions.

When multiple peaks exist for joint distribution differences Δ*p*(*x*, *y*), a homogeneous linear correlation implies that firstly, at least one set of same-signed peaks fall approximately on the same straight line (e.g. Fig. 7e2,e3)); and secondly, anisotropic local distribution difference within each peak should be well-described by the same straight line (e.g. Fig. S7k,l). One can intuitively imagine that the probability of observing three or more peaks to be on the same line in a plane is fairly small. When three or more peaks exist for joint distribution *p*(*x*, *y*) of a BTP, it is likely that corresponding distribution difference Δ*p*(*x*, *y*) has three or more same-signed peaks. Therefore, it is extremely rare for BTPs with three-peak joint distributions fall on the contour line, and we did not observe a single case of BTP with four or more peak joint distributions fall on the contour line. Meanwhile, for a BTP with single or double same-signed peaks in distribution difference, when within-peak distribution is isotropic, one essentially observe approximately homogeneous linear correlations (note that strict homogeneity corresponds to a single straight line with no dispersion of data). However, when one (or both of) same-signed peak(s) are strongly anisotropic, possibility of significant heterogeneous linear correlations start to surface (Fig. 7g1–3)).

It is noted that there are a number of BTPs fall below the contour line in *MI* vs. *r* plots, especially when distances between two participating torsions is below 8 Å (Fig. 6a–d). Generally speaking, there are always some nonlinear contributions to correlation between two torsions unless the joint distribution between them strictly fall on a single straight line. The contour line is simply an effective fit that successfully captures the maximal (minimal) extent of (non-)linear contributions for a larger number of protein BTPs. When a BTP has sufficiently homogeneous linear correlations, it is expected to fall below the contour line, which is an upper bound of mutual information resulted from pure linear correlations for BTPs. This is vividly illustrated by case 6 in *Complex mapping between mutual information and linear correlations* in supporting info, where as two joint distribution peaks gradually increase distances on the line connecting them, the size of each local peak (and heterogeneities associated with it) becomes less and less important, and global homogeneity of linear correlations between them effectively increases. The corresponding position of the DOF pair on *MI* vs. *r* plot fall on the contour line initially and drop below it eventually (Fig. S7j,l).

### Trajectory subset analysis

While these observations are consistent with the idea that either torsional state transitions or strongly anisotropic intra-well torsional motion are necessary for heterogeneity of linear correlations, which seems to be both necessary and sufficient for nonlinear contributions to the overall mutual information. We might not be firmly conclusive regarding the association between torsional state transitions (or strongly anisotropic intra-well torsional motions) and heterogeneity of linear correlations, however. The reason is that for a given protein trajectory set, BTPs fall on the contour line have different identities and physical environment from those locate above it, and there are other differences between two different BTPs in addition to presence/absence of torsional state transitions (or anisotropic intra-well torsional motion). To resolve these uncertainties, we selected some BTPs that manifest strong nonlinear contributions to pair correlations and locate far above the contour line in relevant *MI*-*r* plots from each protein and carried out the following analysis. Firstly, we splitted the original trajectory set into 20 (200 for HEWL due to much larger size of its trajectory set) equally sized subsets. Secondly, both *MI* and *r* were calculated for each of selected BTP on each of the trajectory subsets. For a given BTP, since torsional state transitions occur on specific time scales, we expect to observe various extent of which in different trajectory subsets. Therefore, by observing the extent of nonlinear contributions, relevant torsional DOF distributions and number of joint distribution peaks from trajectory subsets of the same BTP, we effectively excluded the possibility that observed differences are simply due to the fact of observing different BTPs. *MI*-*r* plots of selected BTPs and number of peaks in their joint distributions obtained from trajectory subsets of the four selected proteins were presented in Fig. 8 (see also Fig. S15). Firstly, it is clear that relative importance of linear and nonlinear contributions exhibited in trajectory subsets are widely different from that calculated in the collective set. Secondly, regarding number of peaks for BTP joint distributions and distances to the contour line, very similar pattern was observed as for the corresponding full trajectory sets (Figs 6 and 8). These observations unequivocally confirmed the association between torsional state transitions (or strongly anisotropic intra-well torsional motion) and heterogeneity of linear correlations.

The observed behavior of trajectory subsets is consistent with expectation that torsional state transitions generally occur on relatively longer time scales and are rare events on time scale of snapshots recording (*ps*), and therefore was not observed in many trajectory subsets, for which linear correlations dominate.

## Discussions

### Potential functional relevance of SLR nonlinear BTP correlations and challenges

From a functional point of view, proteins with diverse and significant SLR correlations may be utilized to transmit widely different signals upon different stimuli. It is likely that all hub proteins in protein-protein interaction networks have this property^18^. Conversely, proteins with few SLR correlations may not be versatile in transmitting information over long distances, or at most transmit highly specific and dedicated signals. The biological implication is that for a protein with diverse significant SLR backbone torsional correlations executed by loop residues, potentially rich opportunities exist for designing molecular agent to modulate its functions allosterically. Considering the paramount importance of flexible loop residues in coordinating and participating a wide variety allosteric interactions^5^,^19^,^20^, and the emerging superiority of drug targeting allosteric sites^3^,^4^,^21^,^22^,^23^,^24^, SLR nonlinear correlations exhibited by many *L*-*L* BTPs are of far reaching potential importance in future manipulation of biological systems. However, to fully realize the potential of such versatile SLR, one need to have the capability of predicting such correlations on the one hand, and to understand the mechanism of how information transmit from one site to a distal site in a nonlinear way on the other hand. Both are significant challenges that need to be addressed and are briefly discussed below. Firstly, despite the fact that with steady expected increase of computational power, sub-millisecond to milliseconds MD simulations are expected to be routine in a decade, the fact that we identified SLR nonlinear correlations does not guarantte that we may accurately predict such correlations through extensive MD simulations. The major concern is the quality of force fields in describing such SLR dynamical correlations since we essentially have no reliable reference to perform corresponding optimizations. This is in contrast to the availability of protein data bank for validation of parameters describing individual torsional distributions^25^,^26^. The other possible way is to utilize machine learning technique once we have sufficient reliable data of such SLR nonlinear torsional correlations, which are unfortunately not available for the time being. Secondly, backbone torsions in stable secondary structures mainly exhibit harmonic intra-well dynamics and linear correlations that are on relatively short time scales (nanoseconds or shorter), while nonlinear SLR backbone torsional pair correlations are mainly associated with aharmonic torsional state transitions that occur on much longer and widely different time scales (ranging from tens of nanoseconds up to multiple micron-seconds and beyond as observed in MD simulations). Therefore, if distal nonlinear BTP correlations were indeed transmitted through stable secondary structures, it should not be harmonic vibrational motions that contribute predominantly to linear pair correlations among on-path backbone torsions in corresponding secondary structures. As shown in Figs 4 and 5, significant SLR BTP correlations are predominantly nonlinear. Despite many insightful studies that have been carried out to achieve mechanistic and/or operational understanding of the signal transmission in allostery and to identify on-path communicating residues^21^,^24^,^27^,^28^,^29^,^30^,^31^,^32^,^33^,^34^,^35^,^36^,^37^,^38^,^39^,^40^, the time scale issue remain to be tackled for improved understanding of how SLR nonlinear correlations are transmitted.

### Time scales and spurious correlations

Nonlinear protein BTP correlations, which were demonstrated by our analysis of MD trajectories to occur over large spatial distances, are strong candidates for mediation of allosteric interactions. Significant SLR nonlinear BTP correlations are mainly associated with torsional state transitions, which occur on widely different time scales for different torsional DOFs. It is therefore important to specify time scales when one is interested in correlations of a given BTP. When small MD trajectory sets (up to ~10*ns*) with snapshots interval on *ps* time scales is utilized for analysis, the results are likely to be dominated by strong linear correlations associated with harmonic local motions. Therefore, such analyses are not likely to be insightful for disclosing mechanisms of many functionally important allosteric interactions. Indeed, short time scale linear correlation based network analysis was found to be not effective^41^. A latent problem associated with long time scale correlations is identification of spurious correlations, which remains a grand challenge despite discussions before^14^. For illustration, we constructed *MI* vs. *r* plots for lysozyme based on trajectory sets of different sizes as shown in Fig. 9. One would immediately conclude that a significant fraction of nonlinear correlations observed in Fig. 9b are spurious since they disappear in larger trajectory sets (Fig. 9c–e). However, without larger trajectory sets, we usually have no reliable way of identifying spurious correlations from genuine ones. We may have the following thought experiment. Let’s assume that we have two independent MD trajectories *A* and *B* of the same length for two different proteins, and pick a torsion *a* in *A* and a torsion *b* in *B*, if torsional state transitions of *a* and *b* occur on very similar time scales that happen to be comparable with the total length of the two trajectories, it is likely for us to observe a strong correlation between *a* and *b* if both torsions experienced a small number of torsional state transitions in trajectories *A* and *B* respectively. Such correlation is spurious since we know that there is no physical forces to coordinate torsional state transitions between *a* and *b*, they must go out of phase gradually and eventually lose correlation if the observation was sufficiently long (or sufficiently many observations were made). However, for two DOFs that are in the same protein molecule and we do not have sufficiently long (or many) observation(s), we have no way of differentiating a genuine correlation from a spurious one unless we have the ability to identify physical interaction networks mediating arbitrarily given pairs of DOFs in a molecule, which is just as, if not more, difficult a task itself. We might be tempted to believe that a correlation for a BTP is genuine if we observed many torsional state transitions for the participating torsions. However, we can never be sure that there might be an much slower latent DOF, transitions of which has not been observed but may deminish or strengthen observed correlations for our interested pair. Similarly, we might be tempted to speculate that an observed correlation for a BTP is likely to be spurious if only a few torsional state transitions were observed for participating DOFs, however, such correlations may be equally likely genuine. Fortunately, for many interesting and important biomolecular systems, there are experimental means to estimate time scales of key molecular events, and such time scale information would be of great help in differentiating spurious correlations from genuine ones.

It is noted that the *MI* vs. *r* plot of lysozyme in Fig. 2d is apparently different from Fig. 9e. The difference is that Fig. 2d is generated by taking one out of every ten snapshots available, so a significant weight is given to local torsional motions in calculation of mutual information. While Fig. 9e was obtained by uniformly taking one from every 10,000 snapshots of the same trajectory set, thus essentially the vast majority of local torsional motions were missing. Since in practice, long time scale correlations are more likely to be associated with biomolecular functions, it is suggested that one should focus on global torsional motions.

To be more quantitative on the convergence of distributions of each torsion utilized in correlation analysis, we arbitrarily partitioned trajectory set of each protein into three equally sized subsets, calculated K-L divergence for the distribution of each torsion for each pair of trajectory subsets, and classified the corresponding torsion as satisfactorily converged if the maximum of three K-L divergence value is smaller than 0.2. As shown in Table 2, essentially all torsions are converged for HEWL. For other proteins with smaller trajectory sets, various fractions of torsions are not well converged. Nonetheless, all key conclusions remain the same for converged torsions.

It is important to note that convergence of BTP correlations (or simulation) is dependent upon our interested part of free energy landscape. For example, when our major interest is native conformational transitions, we do not need to observe folding/unfolding events that are likely to be on much longer time scales. A more naive source of spurious correlations is simply unusually small sample size (number of data points), which is highly likely to cause spurious correlations. In this study, we utilized extensive MD trajectories (ranges from a few *μs* to hundreds of *μs*) and a simple random permutation calculation suggest that our results do not suffer from trivial lack-of-data spurious correlations (see Table S2).

## Conclusions

In summary, we analyzed extensive MD simulation trajectories for ten proteins of different sizes and folds, and found that significant SLR nonlinear BTP correlations exist in most of studied proteins. Such nonlinear correlations are predominantly executed by loop residues and mainly associate with aharmonic torsional state transitions of participating torsions, which occur on widely different and relatively long time scales. Alternatively, nonlinear correlations in limited cases may associate with strongly anisotropic local torsional motion. Ultimately, heterogeneous linear correlations of participating torsions are direct causes of significant nonlinear contributions to BTP correlations. In contrast, significant linear correlations are largely limited to shorter spatial range and time scales, and are more likely to associate with residues in stable secondary structures. Long time scales and spatially long range of nonlinear BTP correlations make them strong candidates for mediation of allostery in proteins. Considering the tremendous role of loop residues in participation of biological activities and in transmission of signals, our findings implicate rich possibilities in modulation of biological activities through identification of novel allosteric sites. Meanwhile, time scale difference between SLR nonlinear correlations and local harmonic dynamics warrants further investigations on transmission of allosteric signals across single or multiple protein structural domains. Efforts are undergoing in our group for nonlinear correlation networks of proteins.

## Methods

### MD simulation trajectories for the ten proteins

**Figure 1:**
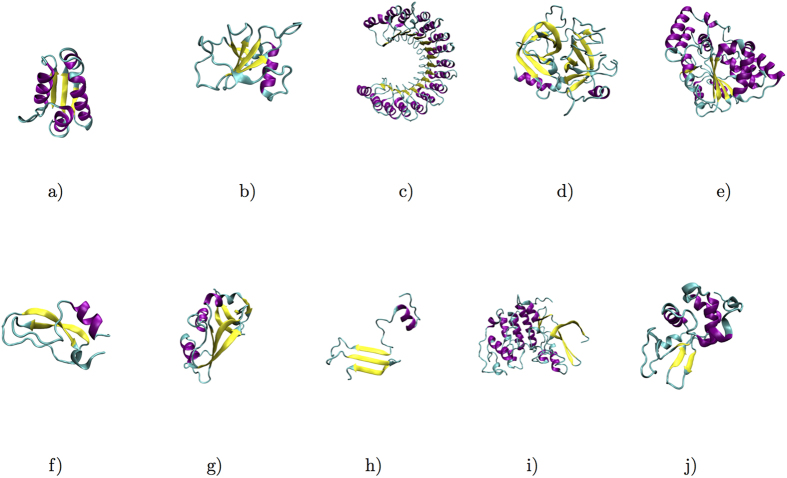
Structures of ten proteins analyzed in this study. (**a**) Barstar (pdb code: 1bta), (**b**) RNase Sa (pdb code: 1rgh), (**c**) Ribonuclease inhibitor (pdb code: 2bnh), (**d**) Kallikrein (pdb code: 2pka), (**e**) Human sulfertrasferase (pdb code: 3f3y), (**f**) Pancreatic trypsin inhibitor (pdb code: 5pti), (**g**) Ribonuclease A (pdb code: 7rsa), (**h**) *β*-barrel-assembly machinery E (BamE), (**i**) Cyclin-dependent kinase 2 (CDK2) and (**j**) Hen egg white lysozyme (HEWL). Hereafter, all proteins are either labeled with its PDB codes or abbreviations shown in parenthesis to be concise. *α* helices are in purple, *β* strands are in yellow, all other secondary structures were termed “loop” in this study and are shown in cyan. This figure was prepared with VMD^48^.

As mentioned in the Fig. 1, pdb codes or abbreviations are utilized to represent names of protein molecules. MD simulation trajectories of 1*bta*, 1*rgh*, 2*pka*, 5*pti*, 7*rsa* and 2*bnh* were taken from our previous studies^42^,^43^. HEWL trajectories were taken from our previous conformational analysis study^44^. Human sulfurtransferase (3*f*3*y*) trajectory set was taken from a previous study^45^ except that we continued to run for 1 *μs* more. *BamE* simulation system was prepared with the same protocol with the initial structure being 2*yh*9. CDK2 trajectory were generated in a similar protocol with these previous trajectories except utilization of the AMBER force fields and package. Briefly, 200 different crystal structures were solvated to generate 200 simulation systems, and a 200*ns* production run were carried our for each simulation system after equilibration, the details of this study is in the process of analysis and will be published elsewhere. A table (S1) was prepared for lengths of various trajectory sets.

### Indexing of backbone torsions

For each protein with *N* residues, 2*N* − 2 backbone torsions were calculated from corresponding trajectory set and utilized for analysis. We did not consider the *ϕ* of the N-terminal residue and *ψ* of the C-terminal residue, and the 2*N* − 2 torsions were indexed by numbers 1 through 2*N* − 2.

### Calculation of circular linear correlations and their instability

Linear correlations between two torsions *x* and *y* were represented using the circular correlation coefficients^10^,^11^,^46^ as shown in following Equations:


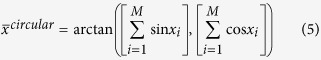



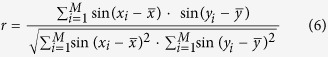


with 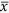 being the average of torsion *x*, *r* being circular linear coefficient and is termed “linear correlation coefficient” in the main text and hereafter, *M* being number of snapshots utilized for analysis, and *x*_*i*_ being the value of *x* for snapshot *i*.

Due to the circular property of torsion angles, brute force calculation of Pearson correlation coefficients is not possible for two torsions. However, when examine carefully the equation utilized for calculating the mean angle (Equation 5), it is apparent that when a torsion has a two peak distribution with 180° difference and approximately equal weights, summation of both sine and cosine terms essentially vanish and the calculated results only reflect local noises. We did observe such instability of circular linear correlations in our analysis, as shown in Fig. S1, three subsets of a BTP with essentially similar joint distribution (*p*(*x*, *y*)) and distribution difference (Δ*p*(*x*, *y*) = *p*(*x*, *y*) − *p*(*x*)*p*(*y*)) have dramatically different calculated linear correlation coefficients, ranging from strongly negative, to essentially negligible and strongly positive linear correlations. Unfortunately, it seems to be difficult to come up with a new formulation that are free of this or other problems for torsions. Therefore, it is essential to be cautious with linear correlation obtained from circular analysis. However, mutual information is free of such instability in addition to the fact that it is linearly related to entropy. It is noted that such instability is more severe with small data sets.

### Calculation of mutual information

Mutual information for each backbone torsional pair (BTP) comprising two torsions *x* and *y* was calculated using the following equations:


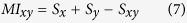



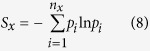



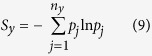



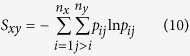


with *S* being informational entropy and *p* being probability in specified bin, *n*_*x*_ and *n*_*y*_ are number of bins utilized for torsions *x* and *y*. In this study, *n*_*x*_ = *n*_*y*_ = 60 if not stated otherwise.

### Distributions of backbone torsions and joint distributions for BTPs

Distributions of each backbone torsion was established by discretizing it into 60 equally sized bins, followed by counting number of snapshot in each bin and normalization with total number of snapshots. Joint distribution of each BTP was established by discretizing the 2*π* × 2*π* square into 3600 equally size squares.

To count the number of peaks in distribution of a single torsion, we first calculated differences between neighboring bins as *δ*_*n*_ = *p*(*n*) − *p*(*n* − 1)(*n* = 0, ···, 59) (note the cyclic property of torsions so we have *δ*_0_ = *p*(0) − *p*(59)). Next we only consider bins with non-zero differences, if we have *δ*_*i*_ < 0 and the first following non-zero *δ*_*j*_ > 0 (*j* ≥ (*i* + 1)), *i* is taken as a boundary bin between two peaks, after all boundary bins were found for a torsion, all bins between two neighboring boundary bins are merged as a collective bin. A collective bin with a probability larger than 0.1 is taken as a distribution peak.

To count the number of peaks in the joint distribution of BTPs, we compare the probability of each bin with that of 8 surrounding bins. A given bin is counted as a tentative distribution peak if its probability is larger than all of the 8 neighbors. Subsequently, if differences between indices of two tentative peaks on both dimension are equal to or smaller than 3, these two tentative peaks will be merged as one joint distribution peak. The reason is that with a 6° torsion bin-size, when indices difference on a given dimension is not more than 3, difference between two peaks are not more than 24° away from each other when they have a index difference not more than 3, and such numerically separate peaks should not be considered as torsional state transitions.

### Torsional distribution convergence analysis

To quantitatively test for convergence of backbone torsional distributions that were utilized in BTP correlation analysis, we first arbitrarily partitioned the trajectory set of each protein into three equally-sized subsets. Subsequently, we calculated K-L divergence for distribution of each backbone torsion for each pair of trajectory subsets and classify the corresponding torsion as satisfactorily converged is the largest K-L divergence is smaller than 0.2. The same binning was utilized for torsions as in the calculation of mutual information.

### Random permutation analysis for sufficiency of statistics in calculation of mutual information and linear correlation coefficient

To test for possible spurious correlation due to lack of statistics (number of data points). We performed correlation calculation based on random permutations. Specifically, in calculation of either *MI* or *r*, in each step *i* for looping through *M* snapshots, we take value of *x* and *y* from independent random snapshots ranging from 1 through *M*. In this way, correlation between *x* and *y* is effectively annihilated and resulting extent of correlation should be due to lack of statistics. The largest spurious correlation value found for all BTPs of each protein were listed in Table S2, since these values are smaller than the size of dots (or other shapes) in relevant plots, no error bar was shown in all plots.

### Inter-torsion distances

The mid point of the central bond is utilized as the position of a given torsion. The inter-torsion distance for a given BTP is defined as distance between positions of its two participating torsions.

### Identity of belonging secondary structures for backbone torsions

Identity of the belonging secondary structure for a backbone torsion is assigned based on DSSP analysis^47^ of the PDB structure utilized to build the simulation system. For CDK2 and HEWL, for which multiple PDB structures were utilized to initiate independent simulations, we carried out DSSP analysis for all utilized PDB structures, and each amino acid was assigned a secondary structure identity that has the most number of observation. In DSSP output, a residue may be assigned one of seven states (*H*: *α* helix, *B*:residue in isolated *β*-bridge, *E*: extended strand, participates in *β* ladder, *G*: 3_10_ helix, I: *π* helix, T: hydrogen bonded turn, *S*: bend). We classified *H* as *α*, *E* as *β* and all others as *L*.

### Represented BTPs in correlation matrices and scattered plots

**Figure 2:**
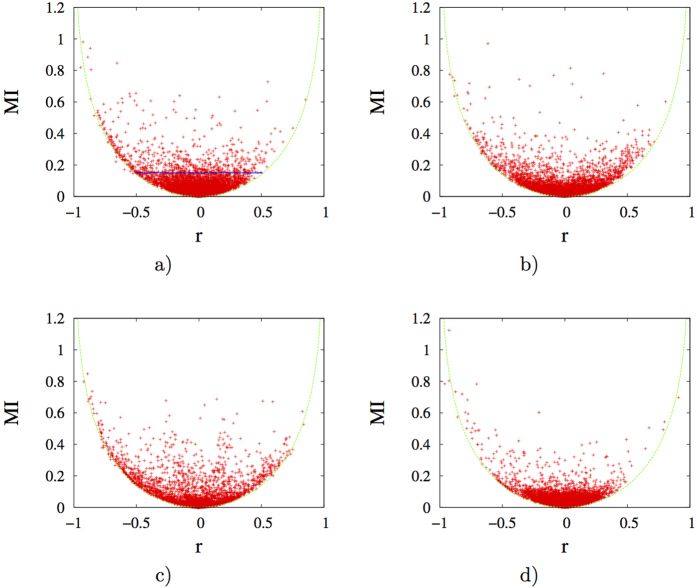
Mutual information *MI* vs. linear correlation coefficient *r* plots for four selected proteins. (**a**) 2*pka*, (**b**) 7*rsa*, (**c**) *CDK*2 and (**d**) *HEWL*. The green dashed line is a universal fit of the contour for data points for all ten studied proteins and is given by Equation 2. The horizontal line in (**a**) is to emphasize that widely different values of linear correlation coefficients may correspond to the same value of mutual information, which is directly related to entropy, and hence free energy of the molecular system. See Fig. S10 for *MI* vs. *r* plots of the remaining six analyzed proteins.

**Figure 3:**
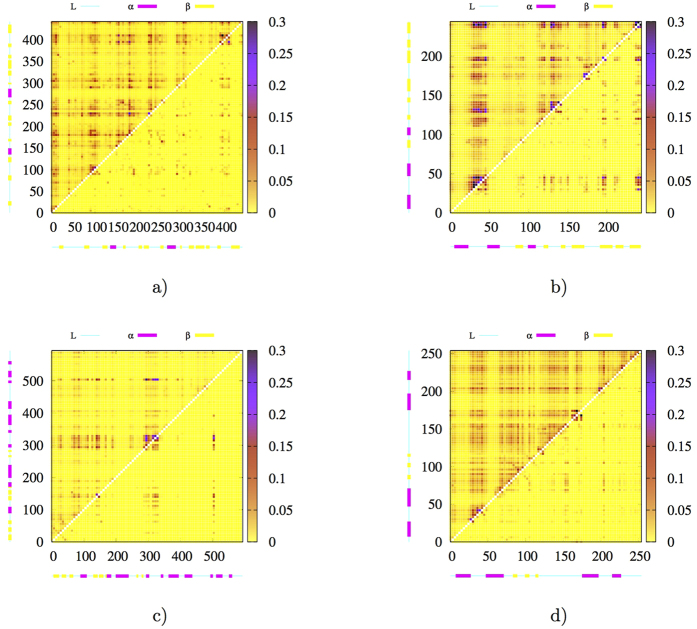
Correlation matrices of four selected proteins, (**a**) 2*pka*, (**b**) 7*rsa*, (**c**) *CDK*2 and (**d**) *HEWL*. For each protein, the full mutual information (MI) is shown in upper-left triangle, and the *MPMI*_*r*_ transformed from linear correlation coefficient *r* is shown in lower-right triangle. The numbers in both horizontal and vertical axis are indices of backbone torsions, which run from N-terminus to C-Terminus. Strength of correlation is indicated by the color bar to the right side. By limiting the range of *MI* (and *MPMI*_*r*_) to [0, 0.3], correlations of BTPs formed by immediate neighboring torsions in sequence were effectively excluded for a better view of correlation patterns elsewhere. See Fig. S11 for *MI* vs. *r* plots of the remaining six analyzed proteins.

**Figure 4:**
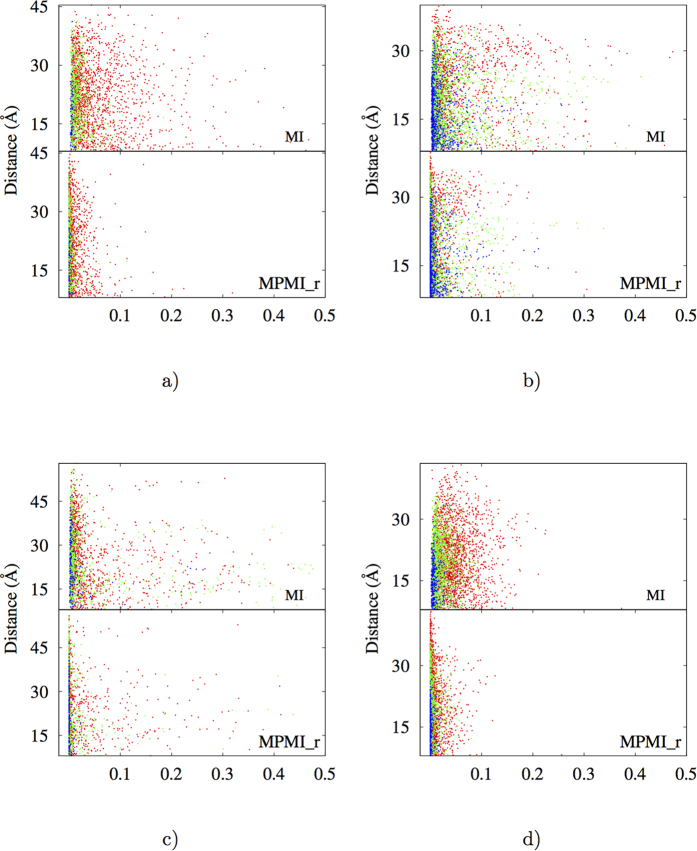
*MI* (top panels) and *MPMI*_*r*_ (bottom panels) for BTPs with various inter-torsion distances (the vertical axis, in Å) of the four selected proteins, (**a**) 2*pka*, (**b**) 7*rsa*, (**c**) *CDK*2 and (**d**) *HEWL*. *α*/*β*-*α*/*β* BTPs are shown in blue, *α*-*L* BTPs in green, and *L*-*L* BTPs in red. See Fig. S12 for similar plots of the remaining six analyzed proteins.

**Figure 5:**
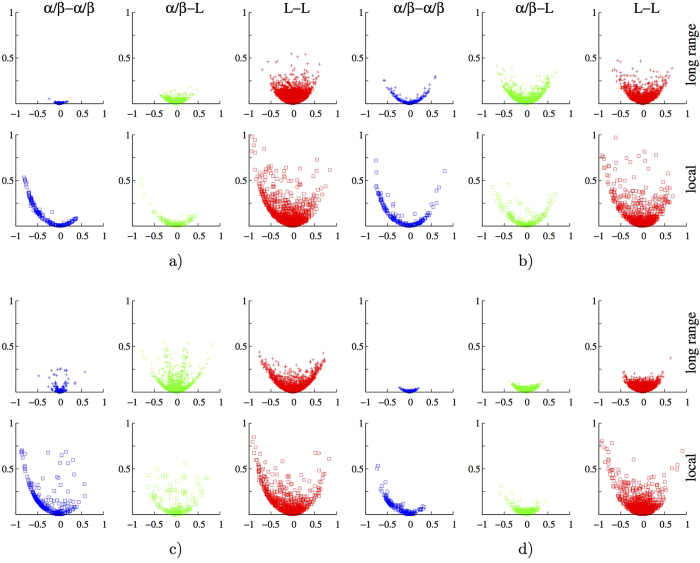
*MI* vs. *r* plots for local (with inter-torsion distances being smaller than or equal to 8 Å, crosses in top panels) and long-range (otherwise, squares in bottom panels) BTPs for four selected proteins, (**a**) 2*pka*, (**b**) 7*rsa*, (**c**) *CDK*2 and (**d**) *HEWL*. *α*/*β*-*α*/*β* BTPs are shown in blue, *α*-*L* BTPs in green, and *L*-*L* BTPs in red. See Fig. S13 for similar plots of the remaining six analyzed proteins.

**Figure 6:**
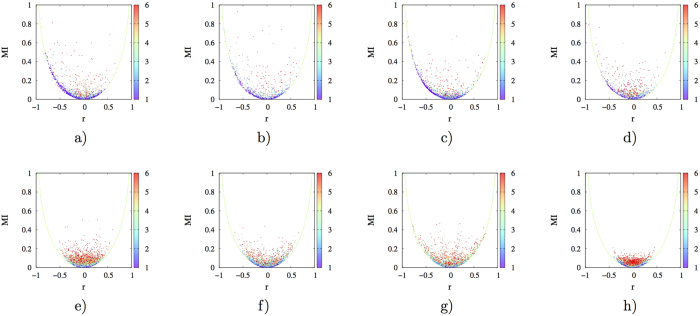
Extent of nonlinear correlations and number of joint distributions peaks (indicated by the side color bar) for local (with inter-torsion distances being smaller than or equal to 8 Å, (**a–d**)) and long range (otherwise, (**e–h**)) BTPs of the four selected proteins. (**a,e**) 2*pka*, (**b,f**) 7*rsa*, (**c,g**) *CDK*2, (**d,h**) *HEWL*. Number of joint distribution peaks is represented by different colors according to the color scale to the right of each plot. See Fig. S14 for similar plots of the remaining six proteins.

**Figure 7:**
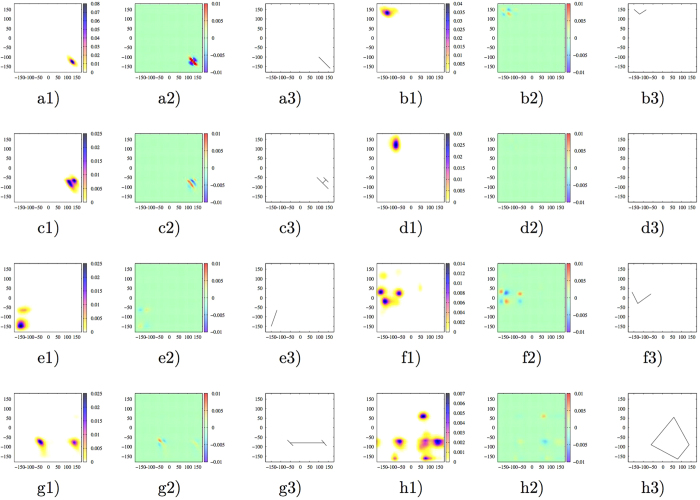
Joint distributions (1), distribution differences (2) and lines connecting positive peaks for distribution differences (3) of eight selected BTPs. (**a**) BTP (4:5) of 1*bta*, a representative BTP with strong homogeneous linear correlations and single peak joint distribution, (**b**) BTP(141:142) of 1*rgh*, a representative BTP with heterogeneous linear correlations and a “L”-shaped anisotropic joint distribution peak, (**c**) BTP (120:121) of 7*rsa*, a representative BTP with heterogeneous linear correlations and an strongly anisotropic joint distribution peak that is essentially two elliptical peaks in immediate contact. (**d**) BTP (9:86) of *HEWL*, a representative BTP with two participating torsions being essentially independent. (**e**) BTP(81:311) of 2*pka*, a representative BTP with strong homogeneous linear correlations and a double-peak joint distribution, (**f**) BTP (141:144) of *CDK*2, a representative BTP with significant heterogeneous linear correlations and six-peak joint distributions. (**g**) BTP (298:299) of *CDK*2, a representative BTP with significant heterogeneous linear correlations and double-peak joint distributions. (**h**) BTP (179:183) of 2*pka*, a representative BTP with significant heterogeneous linear correlations and six-peak joint distributions.

**Figure 8:**
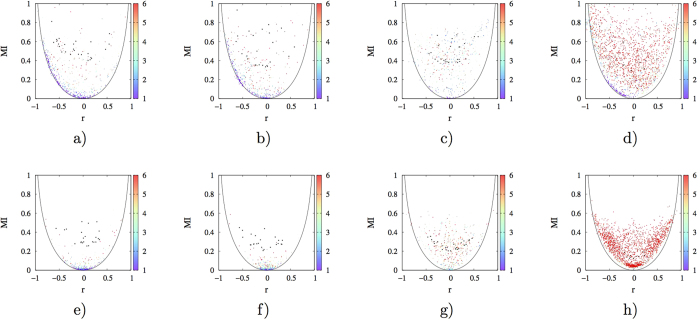
Extent of nonlinear correlations and number of joint distributions peaks (indicated by the side color bar) for local (with inter-torsion distances being smaller than or equal to 8 Å, (**a–d**)) and long range (otherwise, (**e–h**)) BTPs of four selected proteins in 20 (200 for HEWL) equally sized trajectory subsets. (**a,e**) 2*pka*, (**b,f**) 7*rsa*, (**c,g**) *CDK*2, (**d,h**) *HEWL*. Number of joint distribution peaks is represented by different colors according to the color scale to the right of each plot. Black crosses indicate the position of selected BTPs in *MI* vs. *r* plots of the original collective trajectory set. Colored circles are based on calculated *MI* and *r* in trajectory subsets. See Fig. S15 for similar plots of the remaining six proteins.

**Figure 9:**
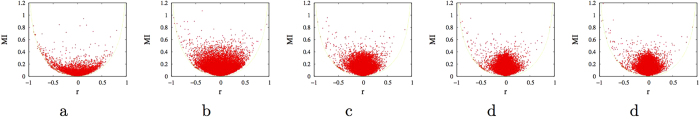
*MI* vs. *r* plots of *HEWL* constructed from trajectory set of (**a**) 1, (**b**)10, (**c**)100, (**d**)1000 and (**e**) 10000 20-*ns* trajectory segments. 5000 snapshots were uniformly taken from corresponding trajectory sets for calculation of *MI* and *r* for all BTPs.

Explicit representation of all BTPs in correlation matrices and various forms of scattered plots would result in excessively large figure files. Only part of BTPs were represented in these plots to avoid this problem while maintaining a satisfactory visualization of the original data, full data sets are available upon requests. Details are stated below: 1) In Figs 3 and S11. A BTP is represented only when indices for both of its participating torsions are multiples of 3 (1*bta*, 1*rgh*, 5*pti*, 7*rsa*, *bame*, *HEWL*), 5 (2*pka*), 7 (3*f*3*y*, *CDK*2) or 11(2*bnh*). 2) In Fig. 2, Fig. S10, Fig. 5, Figs S13 and S14, the following strategies were utilized to reduce figure size. We generated a random number *ran* in the range of [0, *RAN*] for each BTP, which would be selected to be presented in corresponding figures if *MI* > *ran*. for 1*bta*, 1*rgh*, 5*pti*, 7*rsa*, *BamE* and *HEWL*, *RAN* = 0.05, for 2*bnh*, 1*pka* and *CDK*2, *RAN* = 0.1, and for 3*f*3*y*, *RAN* = 0.15. 3) In Figs 4 and S12, we again used the above random number strategy and with *RAN* = 0.03 for 1*bta*, 1*rgh*, 5*pti* and *BamE*, and with *RAN* = 0.5 for 2*bnh*, 2*pka*, 3*f*3*y*, 7*rsa*, *CDK*2 and *HEWL*. In Fig. 9, we used *RAN* = 0.16.

## Additional Information

**How to cite this article**: Long, S. and Tian, P. Nonlinear backbone torsional pair correlations in proteins. *Sci. Rep.* **6**, 34481; doi: 10.1038/srep34481 (2016).

## Supplementary Material

Supplementary Information

## Figures and Tables

**Table 1:** Number (percentage in parenthesis) of BTPs with participating torsions experiencing torsional state transitions in MD trajectory sets of analyzed proteins.

PDB code	Linear BTPs			Nonlinear BTPs			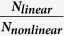
	*N*_*DSP*_ (%)	*N*_*SMP*_ (%)	*N*_*DMP*_ (%)	*N*_*DSP*_ (%)	*N*_*SMP*_ (%)	*N*_*DMP*_ (%)	
1bta	11023 (71.9)	3999 (26.1)	299 (1.9)	3 (3.7)	24 (30.3)	52 (65.8)	193
1rgh	7494 (42.7)	8171 (46.6)	1875 (10.6)	9 (2.1)	70 (16.9)	336 (80.9)	42
2bnh	325998 (78.9)	82284 (19.9)	4992 (1.2)	40 (12.1)	132 (39.9)	159 (48.0)	1248
2pka	53274 (55.2)	37844 (39.2)	5337 (5.5)	27 (1.4)	415 (21.9)	1449 (76.6)	510
3f3y	32589 (22.3)	77234 (52.9)	36105 (24.7)	51 (0.4)	1102 (9.4)	10560 (90.2)	12
5pti	3907 (61.2)	2201 (34.5)	273 (4.3)	9 (15.0)	24 (40.0)	27 (45.0)	106
7rsa	15738 (53.8)	11934 (40.8)	1564 (5.4)	15 (1.6)	170 (18.9)	714 (79.4)	32
bame	2076 (25.6)	4437 (54.7)	1595 (19.7)	4 (0.5)	48 (6.0)	751 (93.5)	10
cdk2	90936 (52.0)	71138 (40.7)	12833 (7.3)	15 (1.2)	171 (14.1)	1028 (84.7)	144
lyzm	9988 (31.7)	15995 (50.7)	5569 (17.7)	23 (2.1)	193 (17.7)	872 (80.1)	29

*N*_*DSP*_: number of BTPs with both torsions having a single peak distribution (Double Single-Peak, DSP). *N*_*SMP*_: Number of BTPs with one of a pair of torsions has multiple (two or more) peak distributions and the other one has a single-peak distribution, (Single Multiple-Peak, SMP). *N*_*DMP*_: Number of BTPs with both torsions having multiple-peak distribution, (Double Multiple-Peak, DMP). A BTP is defined to be nonlinearly correlated when it is vertically above the contour line defined by Equation (2) more than 0.02, or linearly correlated if otherwise. *N*_*linear*_: Number of linearly correlated BTPs; *N*_*nonlinear*_: Number of nonlinearly correlated BTPs.

**Table 2:** Percentage of not well converged torsions (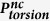) for each analyzed protein trajectory set.

Protein	1bta	1rgh	2bnh	2pka	3f3y	5pti	7rsa	BamE	CDK2	HEWL
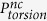 (%)	5.7	17.9	4.9	24.8	28.1	11.4	22.8	49.3	7.7	0.0
