# Supplementary material for: Nonlinear backbone torsional pair correlations in proteins
Source: Sci Rep. 2016 Oct 6;6:34481. doi: 10.1038/srep34481 (PMC5052647; doi:10.1038/srep34481)
Supplement: Supplementary Information [file srep34481-s1.pdf]

Nonlinear backbone torsional pair correlations in proteins

Shiyang Long[†] and Pu Tian[*,†,‡]

School of Life Sciences, Jilin University, Changchun, China 130012, and MOE Key Laboratory of Molecular Enzymology and Engineering, Jilin University, Changchun, China 130012

E-mail: tianpu@jlu.edu.cn

Supporting information

Table S1: Sizes of MD trajectory sets for various proteins (represented by pdb codes or abbreviations). L_{traj}: Length (in nanoseconds) of single continuous MD trajectories, N_{traj}: number of independent MD trajectories. $Interval$: time interval (in picoseconds) for writing MD snapshots.

PDB	1bta	1rgh	2bnh	2pka	3f3y	5pti	7rsa	BamE	cdk2	hewl
$L_{traj}(ns)$	500	500	100	100	1000	100	100	500	200	100
N_{traj}	10	10	10	10	2	10	10	10	200	2000
Interval (ps)	1	1	1	1	1	1	1	1	2	4

[*]To whom correspondence should be addressed

[†]School of Life Sciences, Jilin University, Changchun, China 130012

[‡]MOE Key Laboratory of Molecular Enzymology and Engineering, Jilin University, Changchun, China 130012

Table S2: Maximum of linear correlations ($max - r_{rp}$) and mutual information ($max - MI_{rp}$) calculated for BTPs of various proteins with random permutation.

PDB code	$max - MI_{rp}$	$max - r_{rp}$
1*bta*	0.000336	0.001761
1*rgh*	0.000371	0.001922
2*bnh*	0.001309	0.004622
2*pka*	0.001861	0.004282
3*f*3*y*	0.000930	0.003169
5*pti*	0.001735	0.003684
7*rsa*	0.001840	0.003850
BamE	0.000341	0.001570
*CDK*2	0.000855	0.003103
HEWL	0.000421	0.001896

Table S3: Relative ratio of significantly (mutual information larger than 0.05) correlated SLG BTPs for *L*-*L*, *L*-α/β and α/β-α/β types of all analyzed proteins.

Protein	*L*-*L*(%)	*L*-α/β(%)	α/β-α/β(%)
1*bta*	0.032	0.014	0.000
1*rgh*	3.30	0.11	0.00
2*bnh*	0.378	0.073	0.00
2*pka*	8.83	0.61	0.01
3*f*3*y*	37.9	19.3	10.67
5*pti*	1.74	0.08	0.0
7*rsa*	10.63	4.57	2.28
BamE	22.81	2.05	0.00
*CDK*2	2.44	0.80	0.28
HEWL	1.21	1.13	0.0

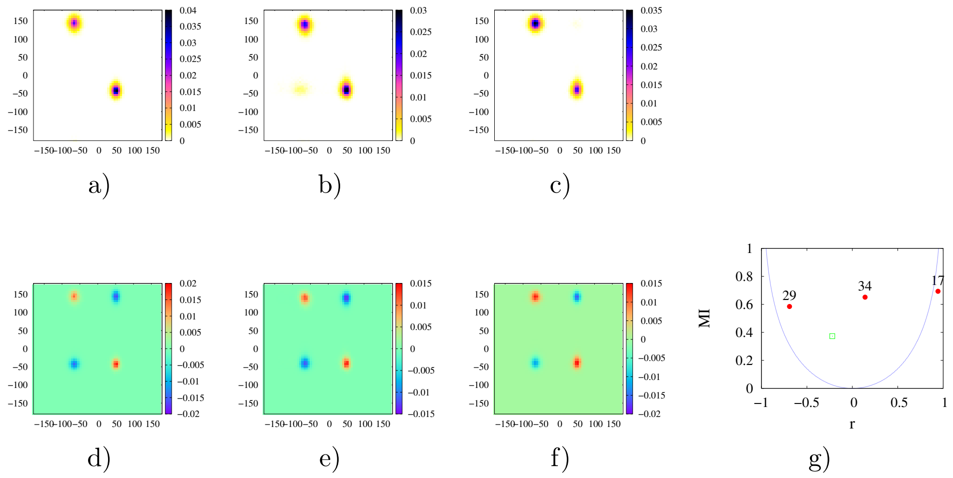

Figure S1: Joint distributions (abc) and corresponding distribution differences (def) for BTP (143,298) of 2pka in selected three out of 40 equally sized trajectory subsets (with indices 17, 29 and 34). a) $p(x_{17}, y_{17})$, b) $p(x_{29}, y_{29})$, c) $p(x_{34}, y_{34})$, d) $\Delta p(x_{17}, y_{17})$, e) $\Delta p(x_{29}, y_{29})$, f) $\Delta p(x_{34}, y_{34})$ and g) the MI vs. r plot, where the calculated mutual information and linear correlation coefficients were shown.

Complex mapping between mutual information and linear correlations

In the main text, we mentioned the lack of thermodynamic support for characterization of correlation with linear correlation coefficients. To provide a better view of complex mapping between mutual information and linear correlations, we calculated both quantities for two variables with artificially constructed distributions and various extent of local and/or global correlations. Specifically, to generate a single peak joint distribution of two independent variable x and y, we first generate a x value according to a gaussian distribution with average being x_0 and standard deviation being σ, followed by generation of a y value according to a gaussian distribution with average being y_0 and standard deviation being σ, and repeat this procedure for n ($n = 200,000$) times. To generate a single peak joint distribution of two correlated variable x and y, we select a parameter a (which is related to the short axis of the resulting ellipse), and use the same procedure to generate a point (x, y) as above, then the distance l between (x, y) and a line through (x_0, y_0) with slope k is calculated, a random

number c in the range of $[0, 1]$ is generated, when $\frac{l}{a} < c$, point (x,y) is taken as a valid point, otherwise it was rejected. After n ($\mathrm{n} = 200,000$) successful points were generated, we will have an effective elliptical joint distribution with long axis having slope k and the short axis related to a. For cases of multiple joint distribution peaks, the total weight is $n = 200,000$, with ratios being specified for each case or equal if not explicitly stated.

Eight various cases of arrangements of such locally independent and/or correlated peaks were constructed to generate different global joint distributions. "Local" correlation/independence is utilized to describe a single -peak joint distribution, while "global" is utilized to describe correlation/independence among multiple joint distribution peaks. Both MI and r were calculated to observe corresponding relationship between these two different ways of correlation characterization. See following figures for MI vs. r plots with the contour line (specified by equation (2) in the main text), joint distributions $p(x, y)$ and difference distributions $\Delta p(x, y) = p(x, y) - p(x)p(y)$ for each case. A common feature observed in all cases is that for strongly homogeneous global linear correlations, corresponding data point fall below the contour line specified by equation (2) in the main text. Considering the fact that data point fall below the contour line is rare (Fig. 2, Fig. 6, Fig. S10 and Fig. S14) in the collective data set, this findings suggest that strongly homogeneous global linear correlations are rare for protein BTPs.

CASE 1: Two equally weighted joint distribution peaks with local independence and global homogeneous linear correlation

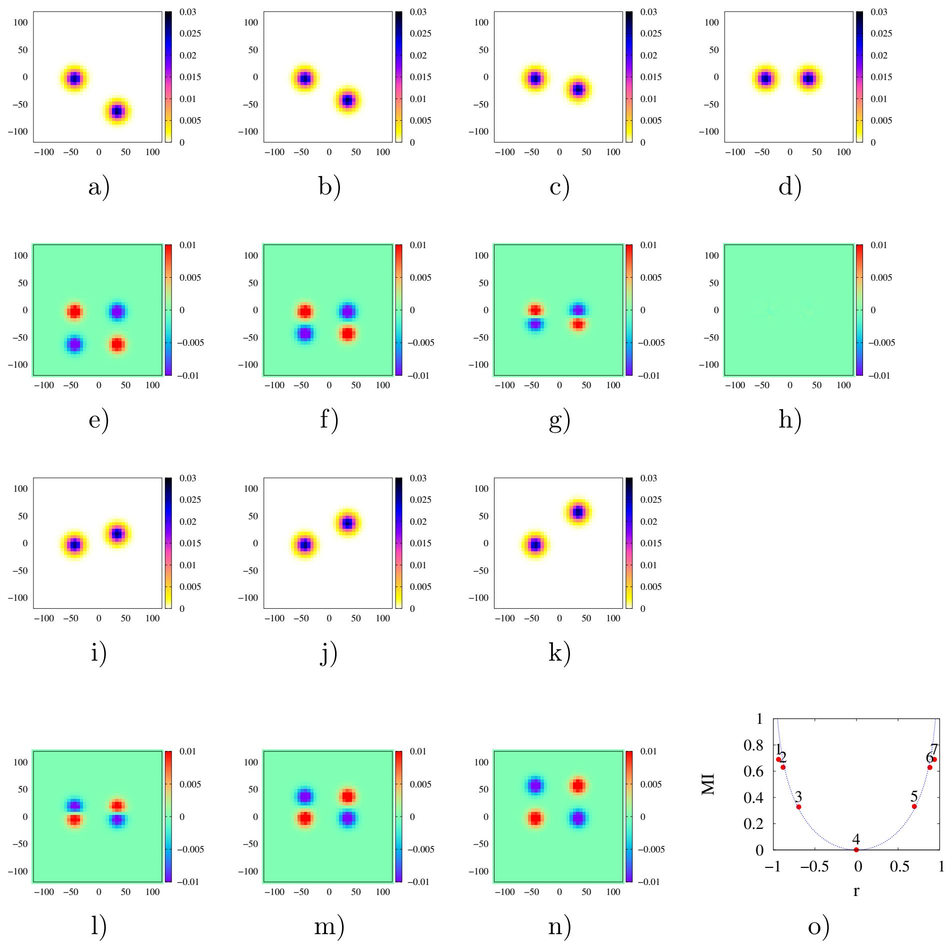

Figure S2: Seven different arrangements of two locally independent joint distribution peaks with different extent of homogeneous global linear correlations. For DOF pairs 1 through 7, the center of the first joint distribution peak is fixed at $(-40, 0)$, and the center of the second joint distribution peak is at (40, -60), (40, -40), (40, -20), (40, 0), (40, 20), (40, 40), and (40,60). Size of both joint distribution peaks is specified by $\sigma = 10$. Joint distributions are shown in a) $p(x_1, y_1)$, b) $p(x_2, y_2)$, c) $p(x_3, y_3)$, d) $p(x_4, y_4)$, i)$p(x_5, y_5)$, j)$p(x_6, y_6)$ and k)$p(x_7, y_7)$. Distribution differences are shown in e)$\Delta p(x_1, y_1)$, f)$\Delta p(x_2, y_2)$, g)$\Delta p(x_3, y_3)$, h)$\Delta p(x_4, y_4)$, l)$\Delta p(x_5, y_5)$, m)$\Delta p(x_6, y_6)$ and n)$\Delta p(x_7, y_7)$. MI vs. r plot is shown in o).

CASE 2: Four rectangularly arranged joint distribution peaks with local independence and approximately homogeneous global linear correlation.

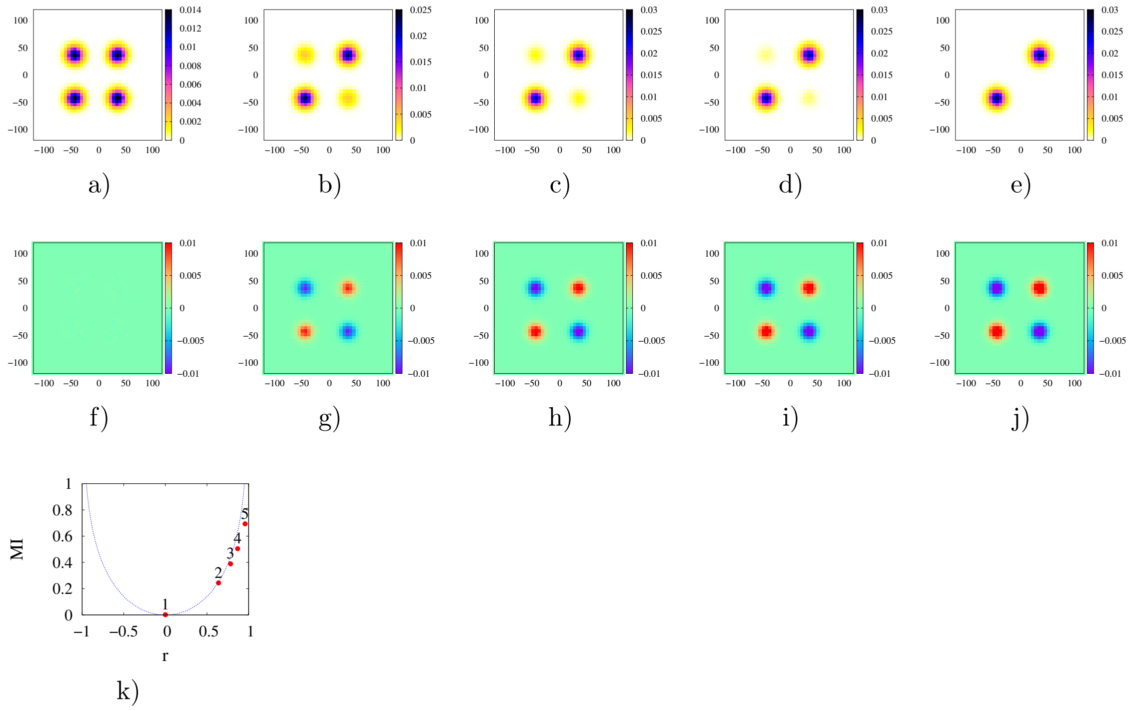

Figure S3: Five different rectangularly arranged locally independent joint distribution peaks with different relative weights, and correspondingly different extent of homogeneous global linear correlations. Centers of the four joint distribution peaks were fixed at $(-40, -40)$, $(-40, 40)$, $(40, -40)$ and $(40, 40)$. The ratio of weights between diagonal and anti-diagonal peaks for the five DOF pairs were $(1 : 1)$, $(5 : 1)$, $(10 : 1)$, $(20 : 1)$ and $(1 : 0)$ respectively. Size of the four joint distribution peaks were specified by $\sigma = 10$. Peaks on the diagonal (or anti-diagonal) direction are equally weighted. Note that DOF pair 5 in this case is the same as DOF pair 7 in *CASE 1*. Joint distributions are shown in a)$p(x_1, y_1)$, b)$p(x_2, y_2)$, c) $p(x_3, y_3)$, d) $p(x_4, y_4)$ and e) $p(x_5, y_5)$. Distribution differences are shown in f)$\Delta p(x_1, y_1)$, g)$\Delta p(x_2, y_2)$, h)$\Delta p(x_3, y_3)$, i) $\Delta p(x_4, y_4)$ and j) $\Delta p(x_5, y_5)$. MI vs. r plot is shown in k).

CASE 3: Seven different cases of single peak joint distributions with homogeneous local linear correlations.

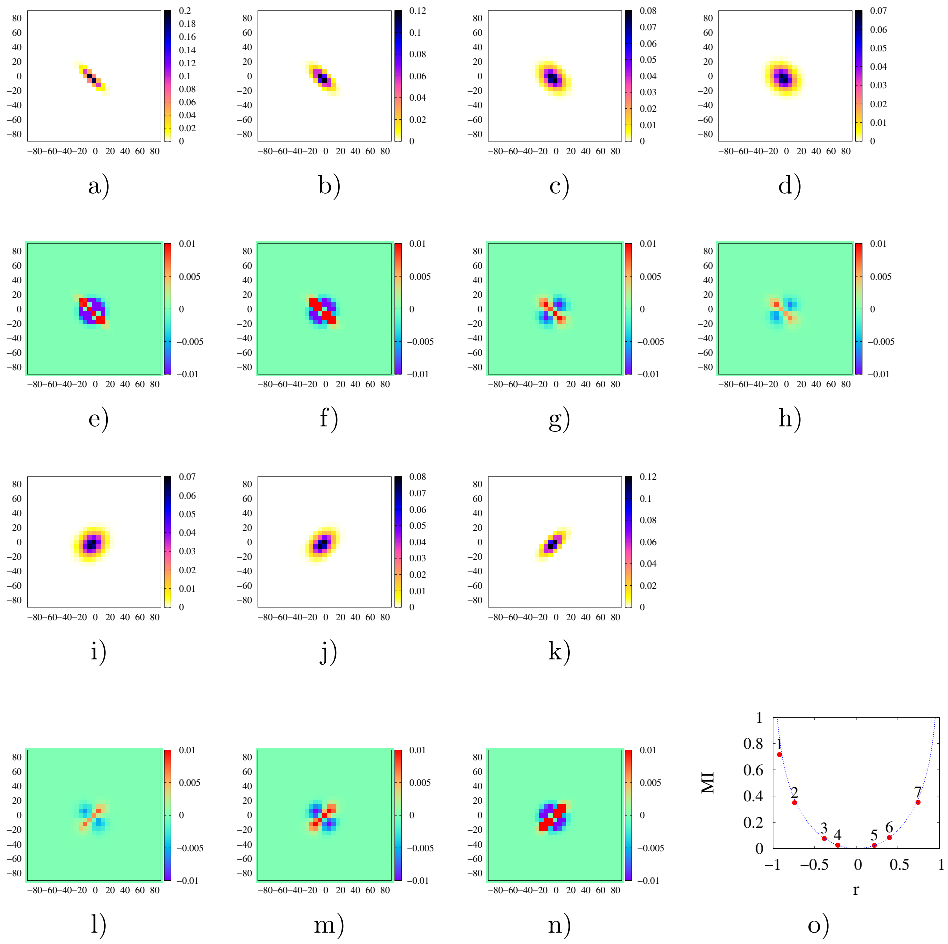

Figure S4: Joint distributions are shown in a) $p(x_1, y_1)$, b) $p(x_2, y_2)$, c) $p(x_3, y_3)$, d) $p(x_4, y_4)$, i) $p(x_5, y_5)$, j) $p(x_6, y_6)$ and k)$p(x_7, y_7)$; Distribution differences are shown in e) $\Delta p(x_1, y_1)$, f) $\Delta p(x_2, y_2)$, g)$\Delta p(x_3, y_3)$, h) $\Delta p(x_4, y_4)$, l) $\Delta p(x_5, y_5)$, m) $\Delta p(x_6, y_6)$ and n) $\Delta p(x_7, y_7)$. (k, a) value for the seven DOF pairs were $(1, 5)$, $(1, 10)$, $(1, 20)$, $(1, 30)$, $(-1, 10)$, $(-1, 20)$ and $(-1, 30)$. MI vs. r plot is shown in o).

CASE 4: Seven different vertical arrangements of two separate elliptical joint distributions with mainly linear local correlations and different extent of globally heterogeneous linear correlations.

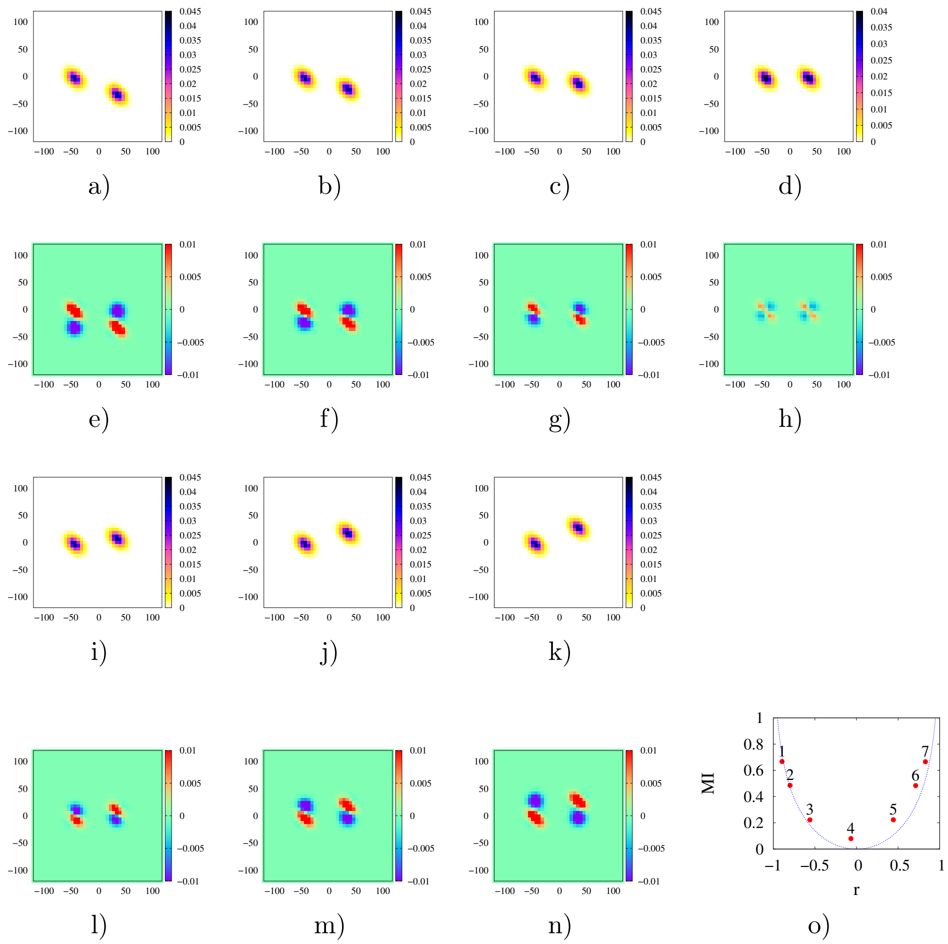

Figure S5: In all seven cases, the orientation and shape of the two elliptical joint distribution peaks were specified by $(k, a) = (-1, 20)$. The center of the first ellipsoidal joint distribution peak was at $(-40, 0)$ for all seven DOF pairs, the centers of the second elliptical joint distribution peaks for the seven DOF pair were $(40, -30)$, $(40, -20)$, $(40, -10)$, $(40, 0)$, $(40, 10)$, $40, 20$ and $(40, 30)$ respectively. Joint distributions were shown in a) $p(x_1, y_1)$, b) $p(x_2, y_2)$, c) $p(x_3, y_3)$, d) $p(x_4, y_4)$, i) $p(x_5, y_5)$, j) $p(x_6, y_6)$ and k) $p(x_7, y_7)$. Distribution differences were shown in e) $\Delta p(x_1, y_1)$, f) $\Delta p(x_2, y_2)$, g) $\Delta p(x_3, y_3)$, h) $\Delta p(x_4, y_4)$, l) $\Delta p(x_5, y_5)$, m) $\Delta p(x_6, y_6)$ and n) $\Delta p(x_7, y_7)$. MI vs. r plot was shown in o).

CASE 5: Six different arrangements of three separate joint distribution peaks with local independence and heterogeneous global linear distributions.

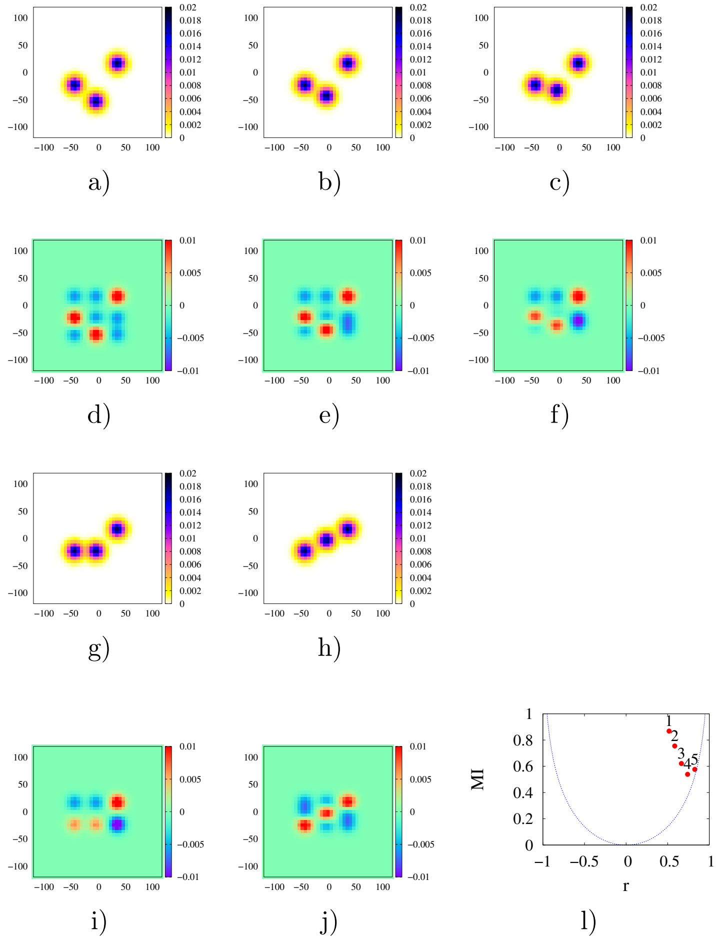

Figure S6: All three joint distribution peaks were equally weighted with size specified by $\sigma = 10$, the center of the first and the third joint distribution peaks were fixed at $(-40, -20)$ and $(40, 20)$ for all six DOF pairs. The second joint distribution peak for the six DOF pairs were $(0, -50)$, $(0, -40)$, $(0, -30)$, $(0, -20)$, $(0, -10)$ and $(0, 0)$ respectively. Joint distributions were shown in a) $p(x_1, y_1)$,b) $p(x_2, y_2)$, c) $p(x_3, y_3)$, g) $p(x_4, y_4)$ and h)$p(x_5, y_5)$. Distribution differences were shown in d) $\Delta p(x_1, y_1)$, e)$\Delta p(x_2, y_2)$, f)$\Delta p(x_3, y_3)$, i) $\Delta p(x_4, y_4)$ and j)$\Delta p(x_5, y_5)$. The MI vs. r plot was shown in l).

CASE 6: Six differently distanced cases of two unevenly weighted elliptical joint distribution peaks with homogeneous local and global linear correlations.

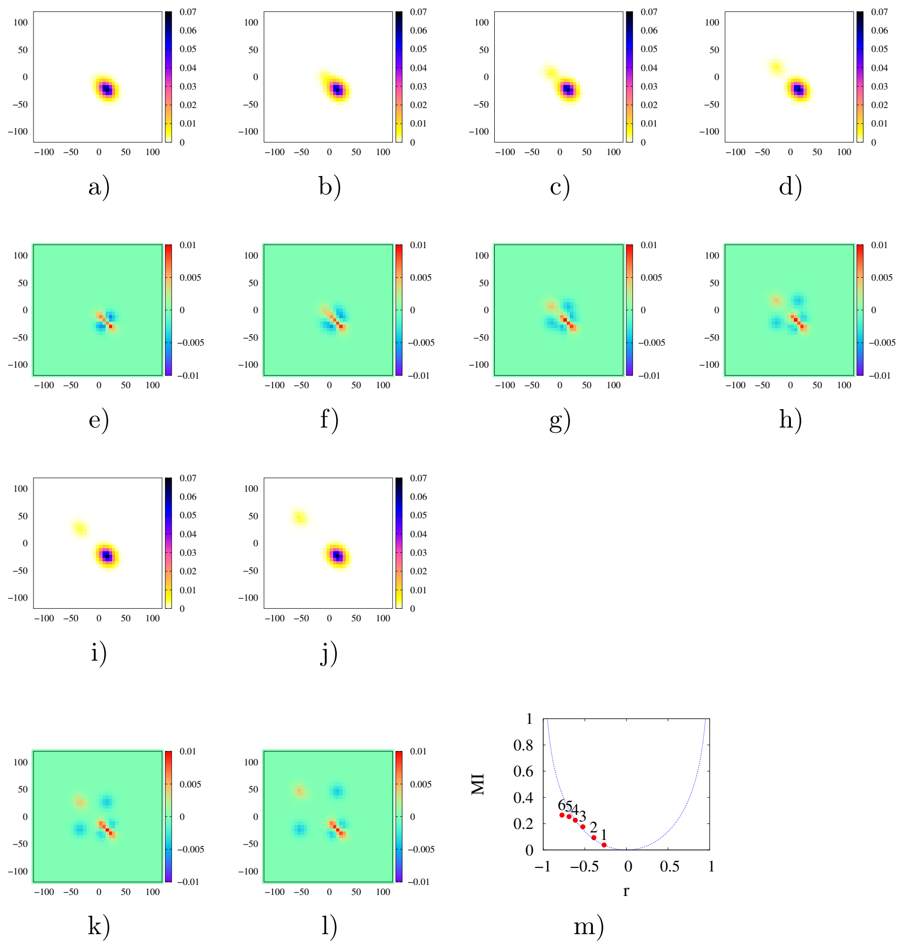

Figure S7: The orientation and shape of both elliptical joint distribution peaks were specified by $(k, a) = (-1, 30)$. The weight ratio between two peaks was 15:1. The center of the first ellipsoidal joint distribution peak was fixed at $(20, -20)$ for all six DOF pairs. The second joint distribution peaks for the six DOF pairs were $(-50, -50)$, $(-40, -40)$, $(-30, -30)$, $(-20, -20)$, $(-10, -10)$ and $(0, 0)$ respectively. Joint distribution peaks were shown in a)$p(x_1, y_1)$, b)$p(x_2, y_2)$, c)$p(x_3, y_3)$, d) $p(x_4, y_4)$, i) $p(x_5, y_5)$ and j)$p(x_6, y_6)$. Distribution differences were shown in e)$\Delta p(x_1, y_1)$, f) $\Delta p(x_2, y_2)$, g)$\Delta p(x_3, y_3)$, h)$\Delta p(x_4, y_4)$, k)$\Delta p(x_5, y_5)$ and l)$\Delta p(x_6, y_6)$. The MI vs. r plot was shown in m).

CASE 7: Ten different arrangements of two separate elliptical joint distribution peaks with heterogeneous global linear distributions.

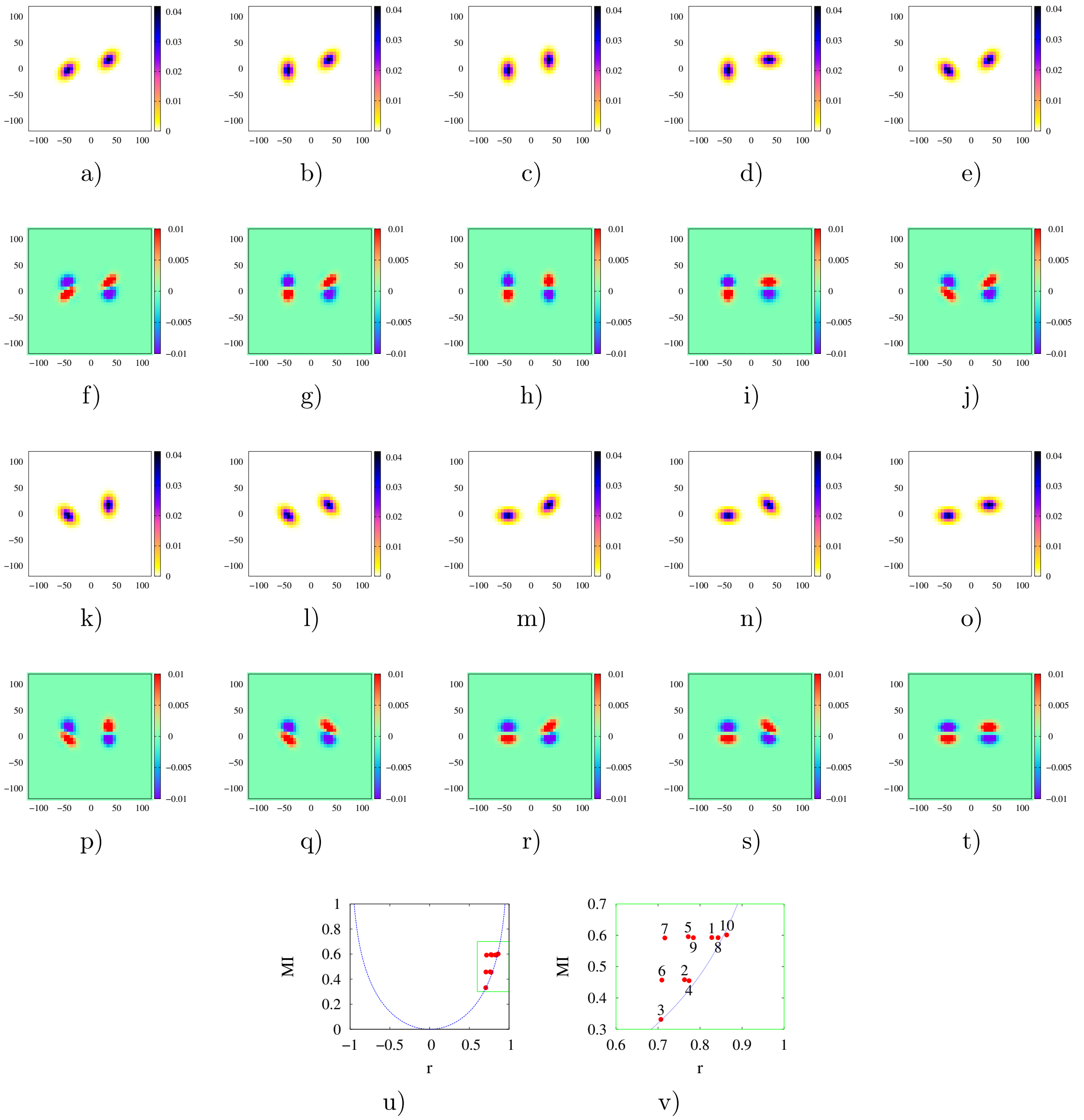

Figure S8: The two equally weighted elliptical joint distribution peaks were placed at $(-40, 0)$ and $(40, 80)$ respectively with the same shape specified by $a = 15$. Orientation combinations of the two peaks were specified by $(k_1, k_2) = (1, 1)$, $(\infty, 1)$, (∞, ∞), $(\infty, 0)$, $(-1, 1)$, $(-1, \infty)$, $(-1, -1)$, $(0, 1)$, $(0, -1)$ and $(0, 0)$, respectively. Joint distributions where shown in a) $p(x_1, y_1)$, b)$p(x_2, y_2)$, c) $p(x_3, y_3)$, d) $p(x_4, y_4)$, e)$p(x_5, y_5)$, k)$p(x_6, y_6)$, l)$p(x_7, y_7)$, m)$p(x_8, y_8)$, n)$p(x_9, y_9)$ and o)$p(x_{10}, y_{10})$. Distribution differences were shown in f)$\Delta p(x_1, y_1)$, g)$\Delta p(x_2, y_2)$, h) $\Delta p(x_3, y_3)$, i)$\Delta p(x_4, y_4)$, j)$\Delta p(x_5, y_5)$, p)$\Delta p(x_6, y_6)$, q)$\Delta p(x_7, y_7)$, r)$\Delta p(x_8, y_8)$, s)$\Delta p(x_9, y_9)$ and t)$\Delta p(x_{10}, y_{10})$. The MI vs. r plot was shown in u) with a local magnification in v).

CASE 8: Four different cases of DOF pairs with a preferred local distribution peak on top of a independent random distribution background.

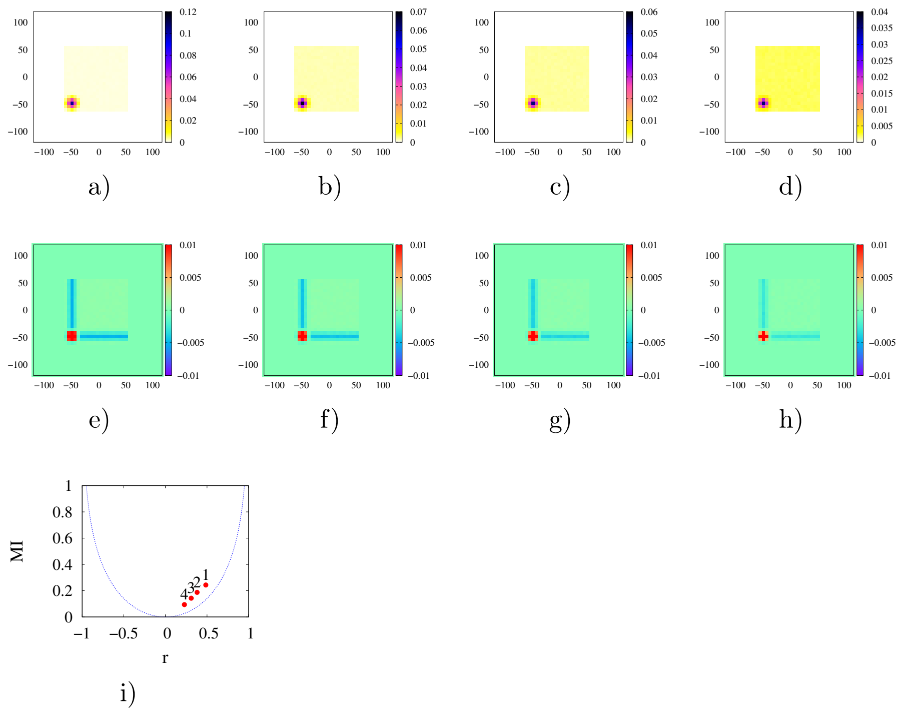

Figure S9: For all four DOF pairs, the preferred peak is posited at $(-45, -45)$ with size specified by $\sigma = 5$, this joint distribution peak is superimposed on a background of uniform joint distribution square specified by $-60 \leq x \leq 60$ and $-60 \leq y \leq 60$. The weight ratio between the preferred peak and the square background are $(1:1)$, $(1:2)$, $(1:3)$ and $(1:5)$ respectively. Joint distributions were shown in a) $p(x_1, y_1)$, b)$p(x_2, y_2)$, c) $p(x_3, y_3)$ and d)$p(x_4, y_4)$. Distribution differences were shown in e)$\Delta p(x_1, y_1)$, f) $\Delta p(x_2, y_2)$, g) $\Delta p(x_3, y_3)$ and h) $\Delta p(x_4, y_4)$. The MI vs. r plot was shown in i).

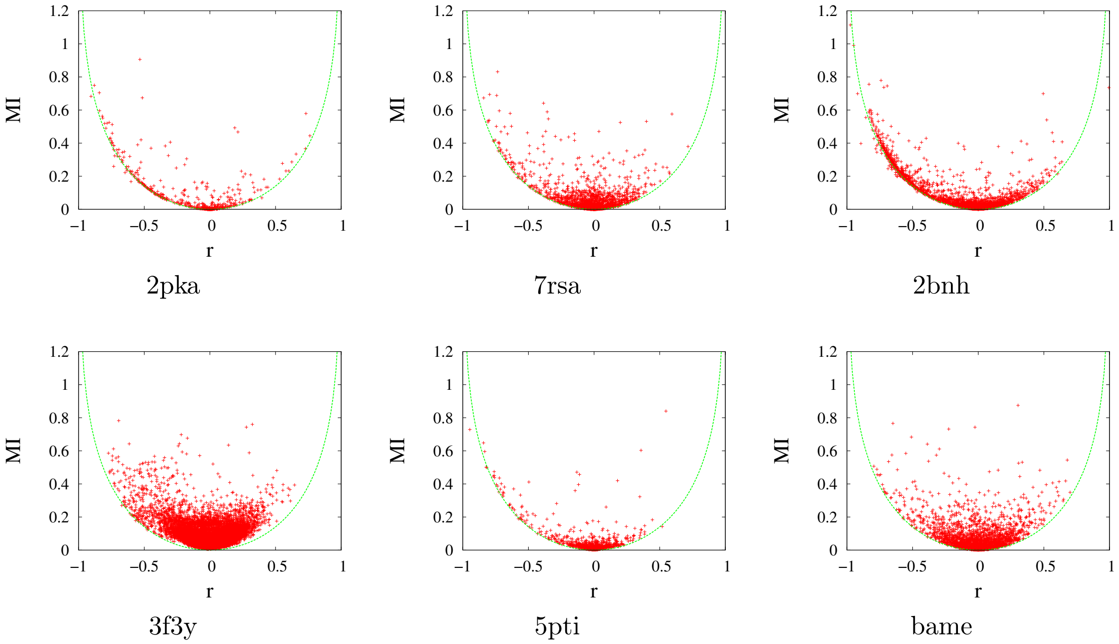

Figure S10: Mutual information MI vs. linear correlation coefficient r plots for six proteins. The green dashed line is a universal fit for all ten studied proteins and is given by equation 2 in the main text.

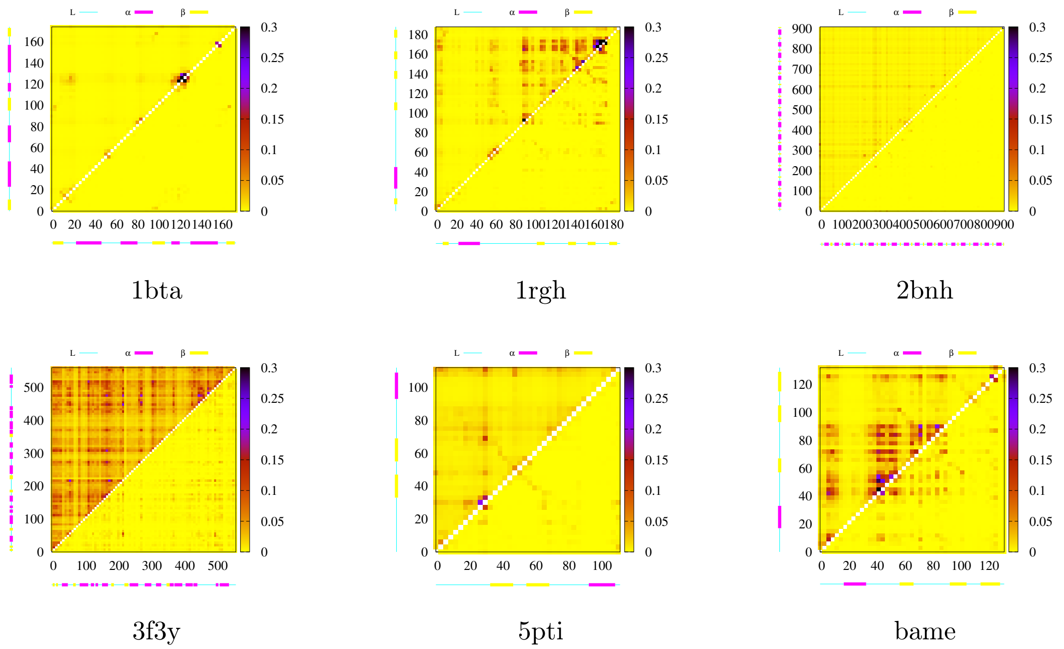

Figure S11: Correlation matrices of six different proteins. For each protein, the full mutual information (MI) is shown in upper-left triangle, and the $MPMI_r$ transformed from linear correlation coefficient r is shown in lower-right triangle. The numbers in both horizontal and vertical axis are indices of backbone torsions, which run from N-terminus to C-Terminus. Strength of correlation is indicated by the color scale to the right side. By limiting the range of MI (and $MPMI_r$) to $[0, 0.3]$, correlations of BTPs formed by immediate neighboring torsions in sequence were effectively excluded for a better view of correlation patterns elsewhere.

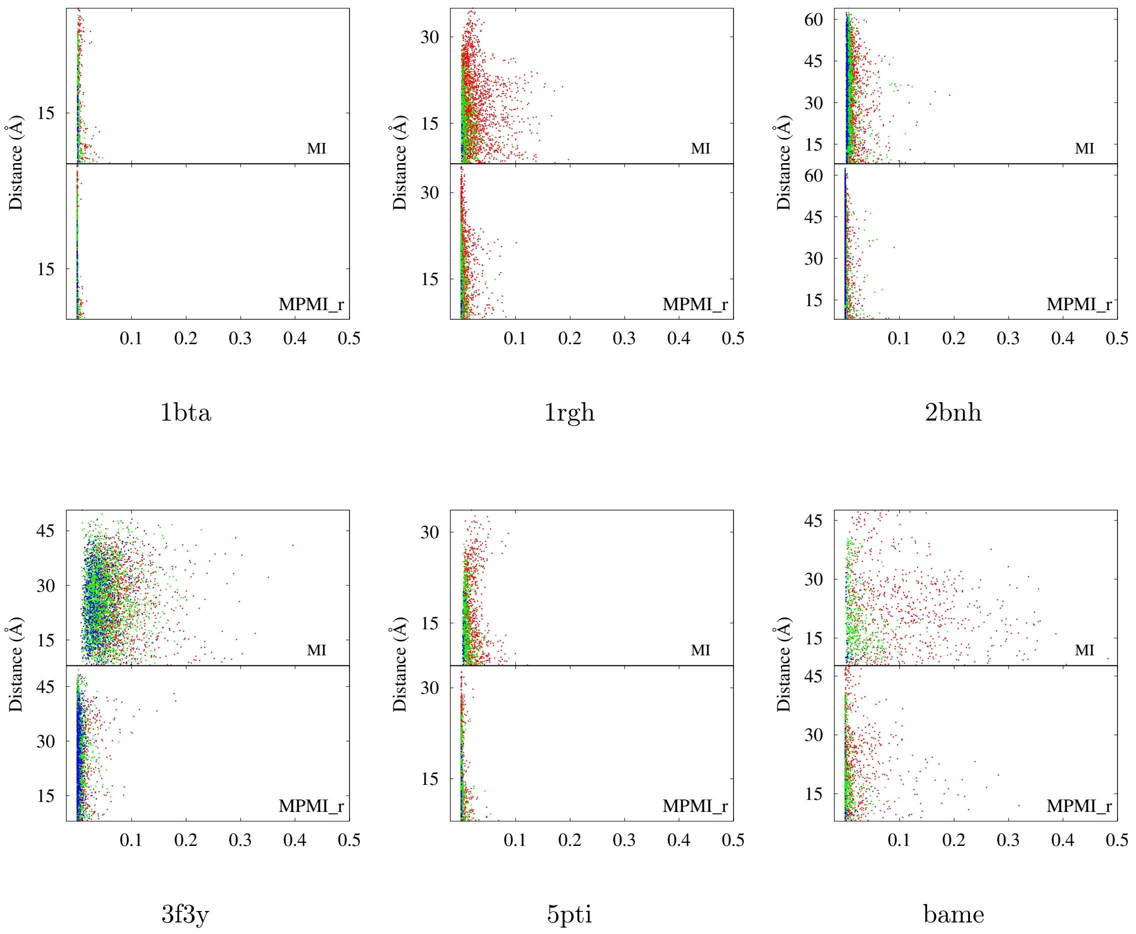

Figure S12: MI (top panels) and $MPMI_r$ (bottom panels) for six analyzed proteins. α/β-α/β BTPs are shown in blue, α-L BTPs are shown in green, and L-L BTPs are shown in red.

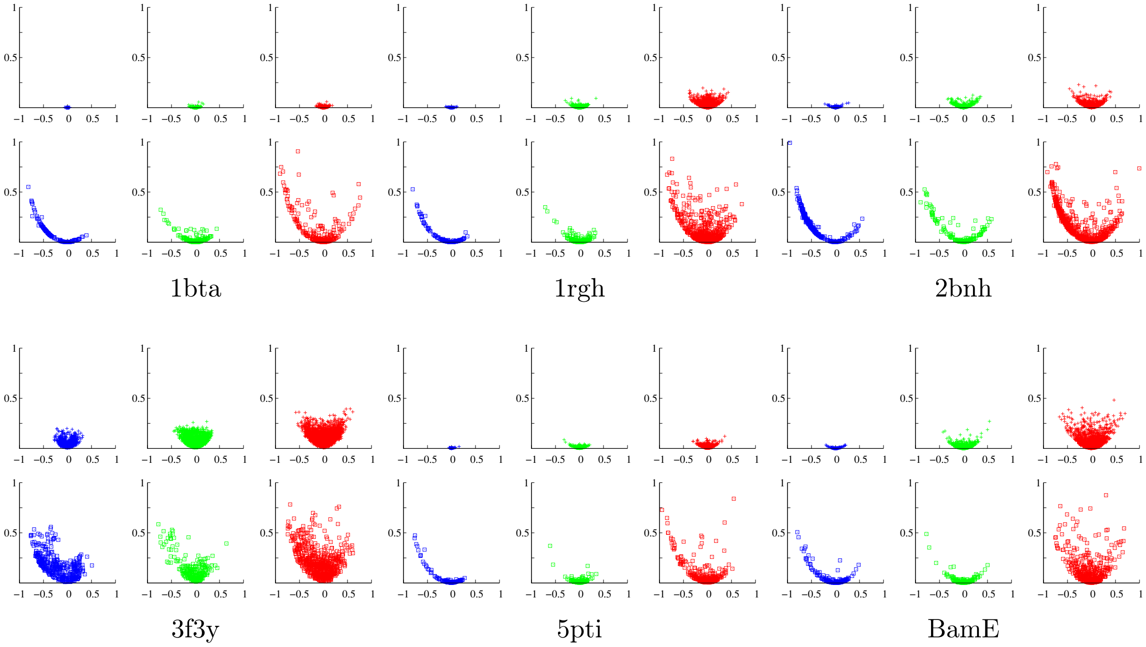

Figure S13: MI vs. r plots for local (with inter-torsion distances being smaller than or equal to 8 Å, crosses in top panels) and long-range (otherwise, squares in bottom panels) BTPs of different types. α/β-α/β BTPs are shown in blue, α-L BTPs are shown in green, and L-L BTPs are shown in red.

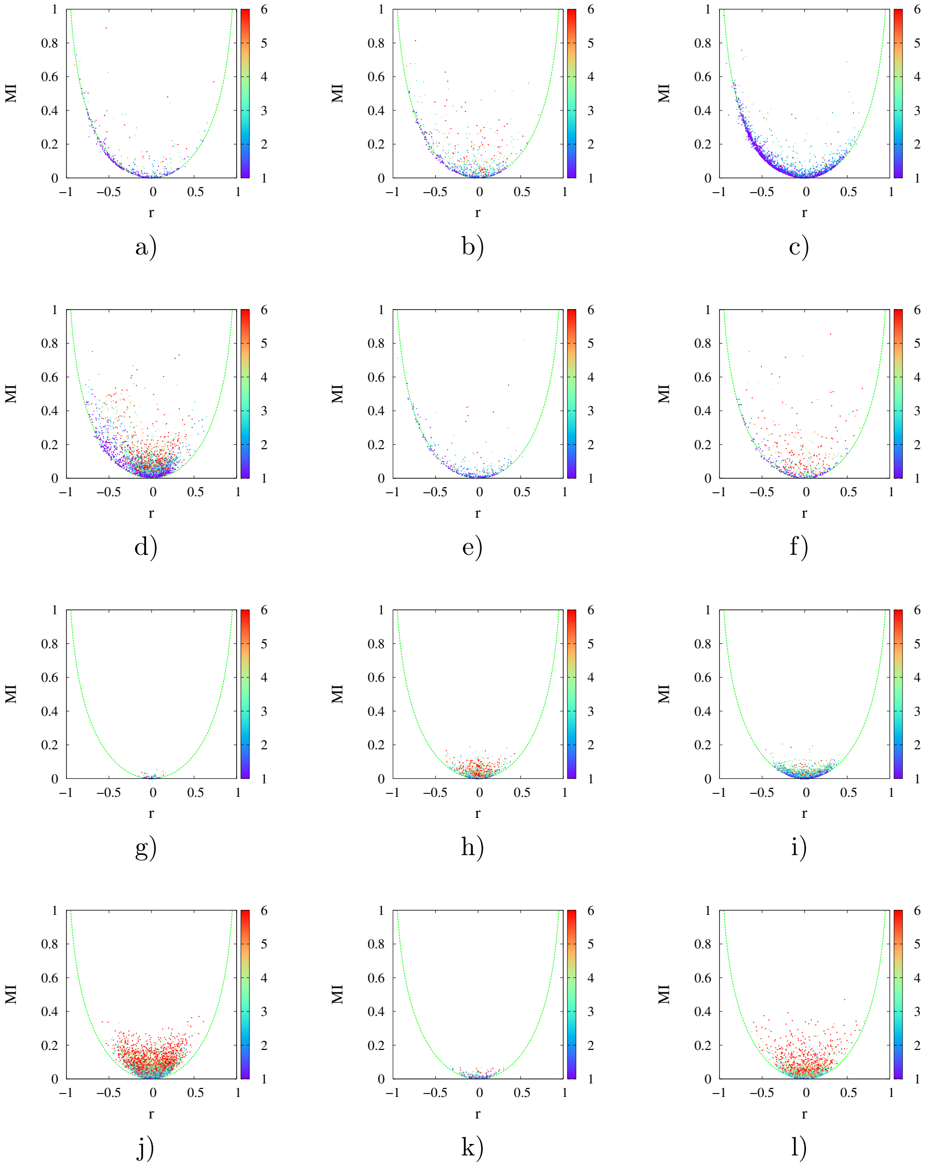

Figure S14: Extent of nonlinear correlations and number of peaks for joint distributions of local (with inter-torsion distances being smaller than or equal to 8 Å, a)b)c)d)e)f)) and long range (otherwise, g)h)i)j)k)l)) BTPs of six analyzed proteins. a) and g) $1bta$, b) and h) $1rgh$, c) and i) $2bnh$, d) and j) $3f3y$, e) and k) $5pti$, f) and l) $BamE$. Number of peaks is represented according to the color scale to the right of each plot.

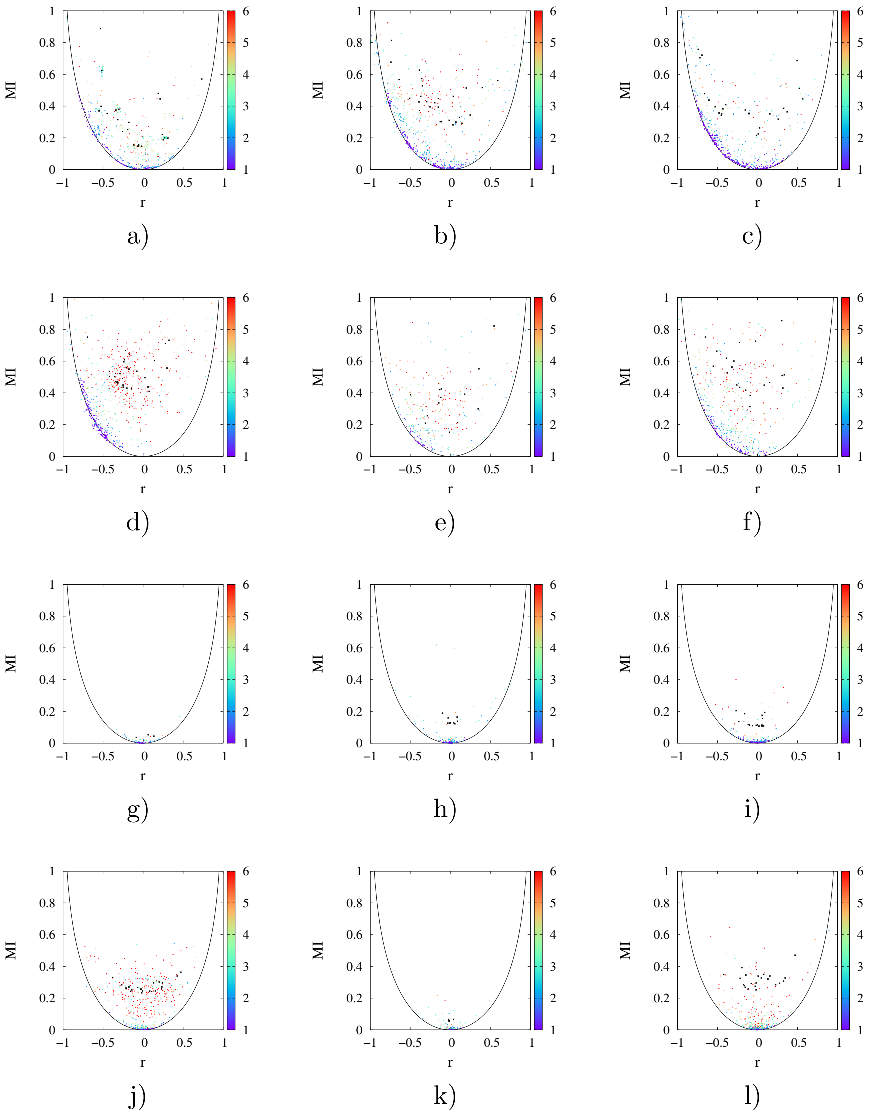

Figure S15: MI vs. r plots for selected local (abcdef) and long-ranged(ghijkl) BTPs calculated in 20 trajectory subsets for six analyzed proteins. a) and g) $1bta$, b) and h) $1rgh$, c) and i) $2bnh$, d) and j) $3f3y$, e) and k) $5pti$, f) and l) $BamE$. Each black cross represents a MI-r pair of a given BTP in the original collective trajectory set. Each circle represents a MI-r pair of a given BTP calculated in one of trajectory subset. Number of joint distribution peaks for BTPs in trajectory subsets are shown in color according to the color scale to the right of plots.
